# A secretion-enhancing *cis* regulatory targeting element (SECReTE) involved in mRNA localization and protein synthesis

**DOI:** 10.1371/journal.pgen.1008248

**Published:** 2019-07-01

**Authors:** Osnat Cohen-Zontag, Camila Baez, Lisha Qiu Jin Lim, Tsviya Olender, Dvir Schirman, Dvir Dahary, Yitzhak Pilpel, Jeffrey E. Gerst

**Affiliations:** Department of Molecular Genetics, Weizmann Institute of Science, Rehovot, Israel; Pacific Northwest Research Institute, UNITED STATES

## Abstract

The localization of mRNAs encoding secreted/membrane proteins (mSMPs) to the endoplasmic reticulum (ER) likely facilitates the co-translational translocation of secreted proteins. However, studies have shown that mSMP recruitment to the ER in eukaryotes can occur in a manner that is independent of the ribosome, translational control, and the signal recognition particle, although the mechanism remains largely unknown. Here, we identify a *cis-*acting RNA sequence motif that enhances mSMP localization to the ER and appears to increase mRNA stability, and both the synthesis and secretion of secretome proteins. Termed SECReTE, for secretion-enhancing *c**is* regulatory targeting element, this motif is enriched in mRNAs encoding secretome proteins translated on the ER in eukaryotes and on the inner membrane of prokaryotes. SECReTE consists of ≥10 nucleotide triplet repeats enriched with pyrimidine (C/U) every third base (*i*.*e*. *NNY*, where *N* = any nucleotide, *Y* = pyrimidine) and can be present in the untranslated as well as the coding regions of the mRNA. Synonymous mutations that elevate the SECReTE count in a given mRNA (*e*.*g*. *SUC2*, *HSP150*, and *CCW12*) lead to an increase in protein secretion in yeast, while a reduction in count led to less secretion and physiological defects. Moreover, the addition of SECReTE to the 3’UTR of an mRNA for an exogenously expressed protein (*e*.*g*. GFP) led to its increased secretion from yeast cells. Thus, SECReTE constitutes a novel RNA motif that facilitates ER-localized mRNA translation and protein secretion.

## Author summary

Proteins destined for secretion from the cell, including soluble secreted and membrane proteins (SMPs), are translocated into the endoplasmic reticulum (ER) either directly upon translation on the ER surface or post-translationally, as in the case of type II membrane proteins. Interestingly, several studies have demonstrated that mRNAs encoding SMPs (mSMPs) are also enriched on the ER, yet how they target this organelle is less clear. The signal recognition particle (SRP), which recognizes N-terminal hydrophobic signals of nascent polypeptides and targets them to an ER-localized receptor, was proposed to mediate the co-translational ER targeting of mRNA. However, more recent studies show that SRP inactivation, as well as the inhibition of translation, do not prevent targeting. Thus, how mSMPs reach the ER and whether the process is translation-independent remain open. Here we identify a *cis*-acting sequence element in mSMPs that appears to facilitate mRNA stability and localization to the ER and, more importantly, enhances protein secretion. This motif, entitled “SECReTE” (secretion-enhancing *c**is* regulatory targeting element) is enriched in nearly all mSMPs in eukaryotes and its addition or removal from mRNAs results in either enhanced or reduced protein secretion, respectively. Thus, SECReTE is a RNA sequence motif that regulates protein translation and secretion.

## Introduction

mRNA targeting and localized translation is an important mechanism that provides spatial and temporal control of protein synthesis. The delivery of mRNA to specific subcellular compartments has a major role in the establishment of polarity in various organisms and cell types, and was shown to be crucial for the proper function of the cell [1,2]. Interestingly, the localization of mRNA is often governed by *cis*-acting elements (“zipcodes”) embedded within the mRNA sequence [1,2]. RNA-binding proteins (RBPs) recognize such sequences and act together with molecular motors to direct the mRNA to its final destination.

The endoplasmic reticulum (ER) is the site of synthesis of secreted and membrane (SMP; secretome) proteins. Moreover, mRNAs encoding for SMPs (mSMPs) are thought to localize to the ER membrane by a distinct translation-dependent mechanism, termed the signal recognition particle (SRP) pathway [3–5]. According to this model, protein translation begins in the cytoplasm and when SMPs undergo translation, a signal peptide present at their amino terminus emerges from the exit channel of translating ribosome and is recognized by the SRP. The SRP then is recruited to its receptor on the ER membrane and translocation of ribosome-mRNA-nascent polypeptide chain complex from the cytoplasm to the ER occurs. There, translating ribosomes interact with the translocon to enable co-translational protein translocation and mRNA anchoring [6,7]. Thus, the SRP model describes mSMPs as components with no active role in the ER translocation process.

However, multiple lines of evidence suggest that there are additional pathways for the delivery of mRNAs to the ER [8,9]. First, attenuation of the SRP pathway did not result in lethality of yeast [10] and mammalian cells [11], and did not have a significant effect upon membrane protein synthesis and global mRNA distribution between the cytoplasm and the ER [11]. Second, genome-wide analyses of the distribution of mSMPs between cytosolic polysomes and ER-bound polysomes demonstrated a significant overlap in the composition of the mRNA in the two fractions and also showed that cytosolic protein-encoding mRNAs are broadly represented on the ER [12–16]. This means that mRNAs lacking an encoded signal sequence or transmembrane domain can also localize to the ER. In agreement with these findings, removal of the signal sequence and the inhibition of translation did not disrupt mSMP localization to the ER [14,17,18]. Third, subsets of secretome proteins are known to localize to the ER in an SRP-independent pathway [19,20]. These proteins are thought to translocate into the ER after translation in the cytosol [21]. In a study that utilized a technique for a specific pull-down of ER-bound ribosomes [22], it was found that there is no significant difference in the enrichment of mRNAs encoding SRP-dependent proteins in comparison to mRNAs encoding SRP-independent proteins on ER membranes. In addition, a subset of ribosomes managed to reach the ER before the emergence of the signal sequence. A possible explanation for these observations could be that mRNAs reach the ER before the ribosomes in an SRP-independent mechanism. If mRNA targeting to the ER does not begin until signal peptide emergence, membrane-bound ribosomes should not be translating portions of the transcript upstream of the signal peptide. However, this was not the case, as translating membrane-bound ribosomes were found to be evenly distributed across entire transcripts in another study [23]. While it is possible that pre-signal sequence-encoding transcripts could arise from translating polysomes, altogether the various findings strongly suggest a scenario whereby mRNAs can localize to the ER prior to translation initiation. Lastly, a recent study demonstrated that conditional SRP depletion from yeast does not necessarily block the co-translational ER targeting of mRNA, especially of those transcripts encoding predicted SRP-independent proteins [24]. Thus, mRNA targeting to the ER likely involves different and, perhaps, multiple paths.

Although it has been difficult to identify *cis*-elements within mRNA that direct it to the ER [25–28], specific sequence characteristics of mSMPs have been identified. For example, sequence analysis of the region encoding the signal sequence revealed a low usage of adenine to create no-A stretches within this sequence [29]. Additionally, mRNAs encoding membrane proteins have a high degree of uracil enrichment, as well as pyrimidine usage, in comparison to mRNAs encoding cytosolic proteins [8,27,30,31]. These findings raise the possibility that the motif resides in a general, more diffuse, fashion within the nucleotide composition of the mRNA molecule.

By examining the sequences of transmembrane domain (TMD)-containing regions in mRNAs, we have now identified high content stretches of pyrimidine (C and U) repeats every third base (*NNY*, *N–*any nucleotide, *Y*–pyrimidine) that can be ≥10 nucleotide triplets in length. Analysis of the transcriptomes of several eukaryotic organisms (*e*.*g*. *S*. *cerevisiae*, *S*. *pombe*, and *H*. *sapiens*), revealed that this sequence pattern is significantly over-represented in mRNAs encoding for secretome proteins, that typically localize to the ER. The location of the motif is not restricted to the coding region but can be present in the untranslated regions (UTRs). Although we originally found the motif by analyzing the sequences of TMDs in secreted membrane proteins, in fact it is enriched at a higher level in mRNAs encoding secreted proteins that lack TMDs. We utilized both computational and experimental tools to establish the existence and significance of this motif. Computational analysis verified that mSMPs are the group most enriched with the motif, while synonymous mutations that either elevated or decreased motif strength (*i*.*e*. number of consecutive pyrimidine repeats) in mRNAs encoding yeast invertase, *SUC2*, as well as cell wall proteins, *CCW12* and *HSP150*, enhanced or reduced protein synthesis and secretion, respectively. This motif, which appears to facilitate mRNA stability, localization and translation at the ER, we have named the secretion-enhancing *c**is* regulatory targeting element (SECReTE). Importantly, we show that SECReTE is enriched in secretome-encoding transcripts in all organisms examined, from prokaryotes to both lower and higher eukaryotes. This suggests that SECReTE may have a conserved role in the translational control of mRNAs either as a targeting motif or in other processes such as translation efficiency, mRNA processing (*i*.*e*., polyadenylation, capping, splicing), mRNA decay, and secondary structure, *etc*. We propose that SECReTE is important, not only to understand how mRNAs may reach the ER in eukaryotes, but may have practical applications in the field of biotechnology.

## Results

### Identification of a pyrimidine repeat motif in mRNAs encoding yeast secretome proteins

RNA codons for the major hydrophobic residues (*e*.*g*. valine, isoleucine, leucine, methionine, and phenylalanine) are enriched in uracil in their second position [31], although others like proline, alanine, serine and threonine, which can also be found in TMDs, contain cytosine at this position. We speculated, therefore, that triplet repeats of the form, *NYN* (where *N =* any nucleotide and *Y* = pyrimidine—U or C), might be common to proteins destined for translation at the ER (*i*.*e*. secreted and membrane proteins). We further speculated that uninterrupted repeats of this nature might be indicative of an RNA localization motif that could, potentially, exist in any frame. Thus, we examined mRNAs encoding yeast secretome proteins for the presence of consecutive pyrimidine repeats every third nucleotide (*i*.*e*. *YNN*, *NYN*, or *NNY*) in both the coding and UTR regions, using computational analysis.

**Fig 1:**
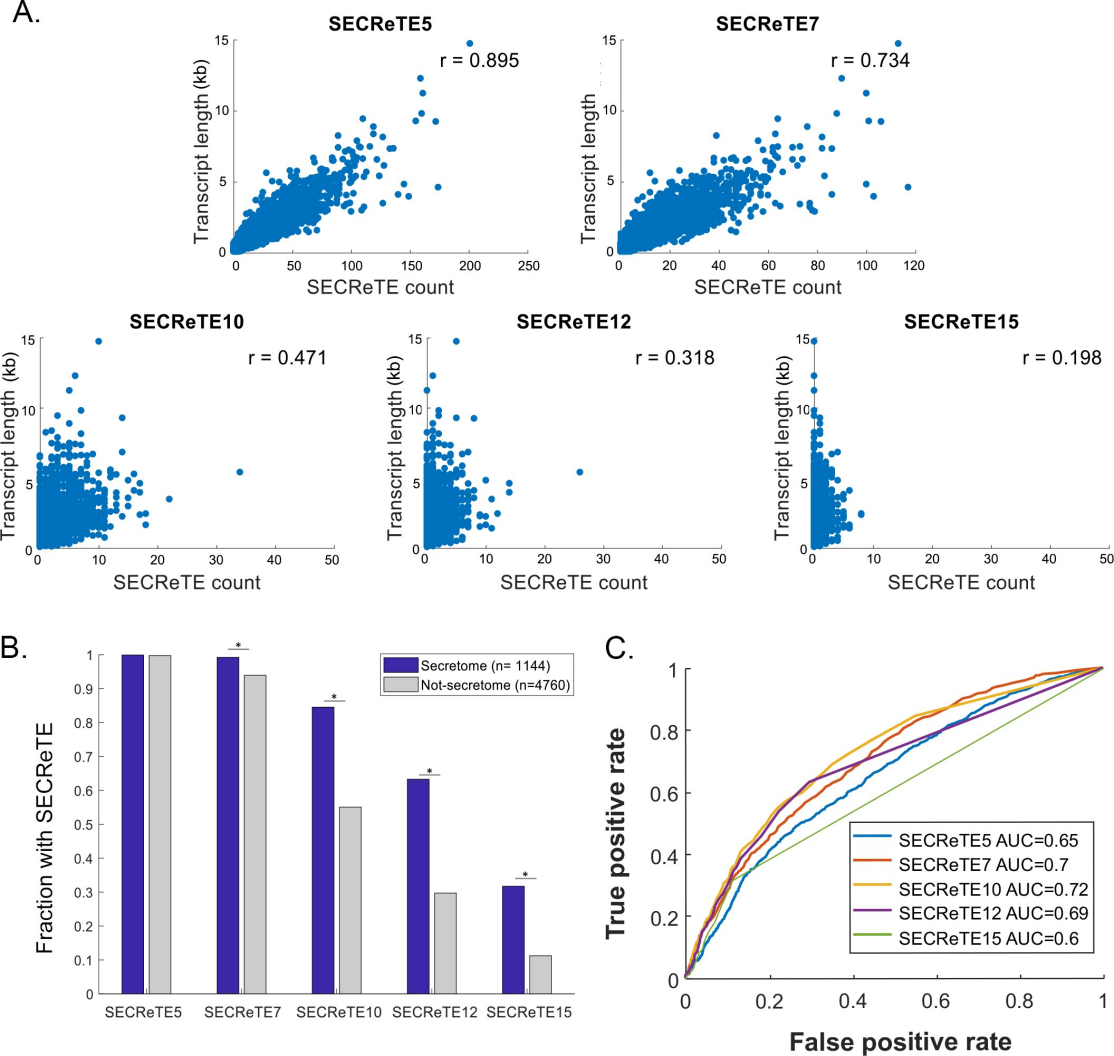
Determination of the number of *NNY* repeats to use as a threshold for SECReTE. **(A) Correlation between SECReTE count and transcript length.** The total SECReTE count was calculated for the coding region of each yeast gene (5904 genes scored) by counting the number of consecutive *NNY* repeats present in the transcript sequence according to the indicated threshold, and in all three frames. Scatter plots represent the correlation between the SECReTE count and gene length. The SECReTE count does not correlate with gene lengths above a threshold of 10 *NNY* repeats (SECReTE10). *r* represents the Pearson correlation coefficient. (**B) SECReTE motifs are more abundant in the mRNAs coding for secretome proteins than for non-secretome proteins.** SECReTE presence, according to the indicated threshold, was counted in mRNAs coding for secretome (blue) and non-secreted (gray) proteins. Bars represent the fraction of SECReTE positive transcripts at the indicated threshold. SECReTE abundance is significantly higher in secretome mRNAs. *p ≤ 2.28E-13, Chi-squared test. (**C) SECReTE10 maximizes the ability to distinguish secretome transcripts.** ROC curves were plotted for each of the indicated thresholds. Secretome transcripts were used as the “true positive” set, while non-secretome transcripts were used as the “true negative” set. The AUC (area under the curve) of SECReTE10 was the highest.

We first determined how many such repetitive nucleotide triplets might best differentiate mSMPs from non-mSMPs (*i*.*e*. other nuclear-encoded mRNAs). For that, the number of consecutive triplet repeats along an mRNA transcript was scored according to a defined threshold (*e*.*g*. 5, 7, 10, 12, and 15 repeats). For a random motif we expected to see a linear correlation between the probability of its appearance(s) in a gene with gene length, as shown in Fig 1A. We examined SECReTE lengths between 5 and 15 (*e*.*g*. nucleotide triplet lengths of 5,7,10,12, and 15) and indeed observed a direct correlation between SECReTE count and gene length for SECReTE5 and SECReTE7 (Fig 1A). However, the dependency on gene length becomes significantly weakened for SECReTE10 and above, where motif occurrence at ≥10 triplets correlates poorly with gene length (Fig 1A). This implies the presence of ≥10 consecutive repeats is not a random phenomenon and may be important.

If SECReTE repeats ≥10 (*e*.*g*. termed here as “SECReTE10”) play a role in protein secretion, we expect them to be more abundant in mRNAs encoding secretome proteins, as defined according to Ast *et al*. [19]. To test this possibility, we divided the complete yeast genome into two gene sets: secretome and non-secretome, and calculated the fraction of transcripts that contain at least one instance of SECReTE per gene in each gene set. We found transcripts coding for secretome proteins are enriched with SECReTE motifs of length >7 (Fig 1B), in comparison to transcripts encoding non-secretome proteins. To test the motif length that gives the most significant separation between secretome and non-secretome transcripts, we evaluated the different thresholds for their ability to classify mSMPs using receiver operator characteristics (ROC) analysis. *Bona fide* secretome protein-encoding transcripts were used as a true positive set and non-secretome protein-encoding transcripts were defined as true negatives. As seen (Fig 1C), motifs with ≥10 repeats (*i*.*e*. SECReTE10 and above) maximally differentiated (in terms of ROC area under the curve) secretome transcripts from non-secretome transcripts. As the occurrence SECReTE10 did not show a dependency upon gene length and gave the most significant separation between secretome and non-secretome transcripts, we used it as the threshold by which to define motif presence in subsequent analyses. Previous studies have used high throughput analyses to quantify the level of enrichment of transcripts on yeast ER-bound ribosomes and ER membranes [22,23]. By comparing the cumulative distribution of the ER enrichment value of SECReTE10-containing transcripts to transcripts lacking SECReTE10, we could verify that a higher fraction of SECReTE10-containing transcripts is indeed enriched on ER-bound ribosomes (S1A and S1B Fig) and ER membranes (S1C Fig). In contrast, SECReTE10-containing transcripts are not enriched on mitochondrial ribosomes, in comparison to transcripts lacking SECReTE10 (S1D Fig).

### SECReTE abundance in mSMPs is not dependent on the presence of a TMD

**Fig 2:**
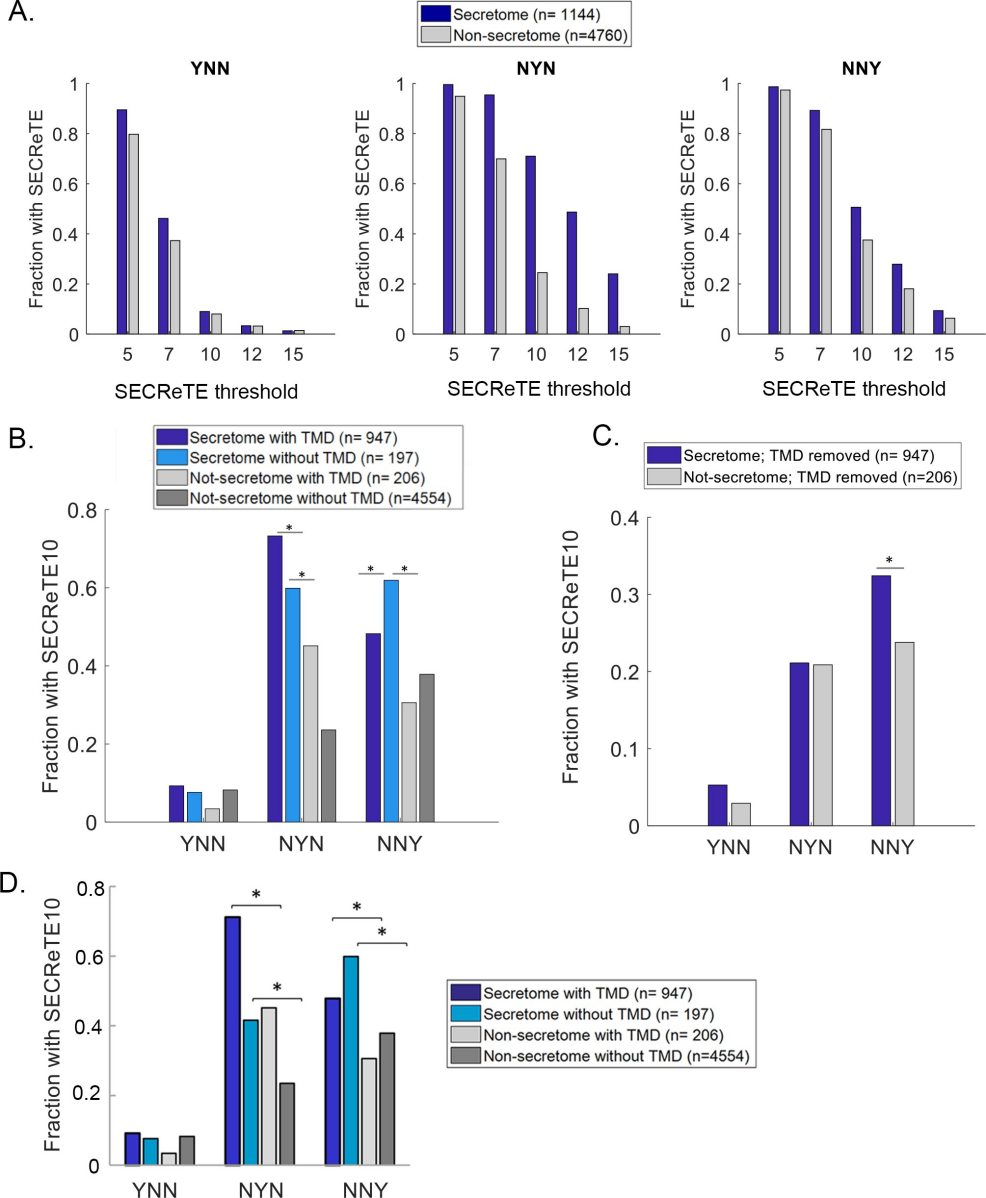
SECReTE abundance in mSMPs is TMD-independent. **(A) SECReTE is abundant in the second position of the codon.** SECReTE abundance was calculated for each codon position separately. SECReTE abundance in mSMPs is most significant in the second codon position (*NYN*), but significant differences were also detected in in the third position (*NNY*). *p≤ 9.9E-10, Chi-squared test. **(B) SECReTE is also highly abundant in the mRNAs encoding soluble secretome proteins.** SECReTE10 presence was examined separately for TMD-containing proteins and soluble secreted proteins. A higher fraction of mRNAs coding for soluble secreted proteins (Secretome without TMD; cyan) contains SECReTE in comparison to non-secretome transcripts, either with or without a TMD (Non-secretome with TMD; dark gray, Non-secretome without TMD; light gray). In the third codon position (*NNY*), the fraction of soluble secreted proteins is even larger than TMD-containing secretome proteins and is significant. *p≤3.03E-3, Chi-squared test. **(C) SECReTE is abundant at the third position after removal of the TMD sequence.** SECReTE10 presence was scored in mRNAs coding for membrane proteins after the encoded TMD was removed. SECReTE10 is significantly more abundant in the third position (*NNY*) in mRNAs encoding secretome proteins (blue) than non-secretome proteins (gray), even after removal of the TMD sequence. *p = 0.01, Chi-squared test. **(D) SECReTE is highly abundant in mSMPs after the removal of the SSCR sequence.** SECReTE10 presence in the different positions was scored after the regions encoding signal peptides were removed. Similar to the results shown in *B*, mRNAs coding for soluble secreted proteins (cyan) are enriched with SECReTE, in comparison to non-secretome transcripts without a TMD (light gray). In the third codon position (*NNY*), the fraction of soluble secreted proteins is larger than for TMD-containing secretome proteins and is significant. *p≤5.93E-3, Chi-squared test.

To ascertain whether SECReTE enrichment in mSMPs is not merely due to its presence in encoded TMDs, we determined at which position of the nucleotide triplet in SECReTE10 elements is the pyrimidine (*Y*) is located: *i*.*e*. first (*YNN*); second (*NYN*); or third (*NNY*). We calculated SECReTE10 abundance separately for each position using only the coding sequences (*i*.*e*. from start codon to the stop codon; CDS) and without the UTRs. While the signal is present in the second position (Fig 2A; *NYN*), as expected, it is also abundant in the third position of the codon (Fig 2A; *NNY*). In contrast, the SECReTE10 element is poorly represented in the first position, *YNN* (Fig 2A). Importantly, the abundance of *NNY-*based motifs suggests that the TMD is probably not the only element that confers SECReTE enrichment in the coding sequences of yeast mSMPs. A list of all SECReTE motifs (*i*.*e*. ≥10 repeats, either *NYN-* or *NNY-*based) present in the coding sequences of the yeast genome and their position within the mRNA is listed in S3 Table.

Next, we checked for the presence of SECReTE10 in mRNAs coding for TMD-containing proteins and soluble secreted proteins separately. As expected, more transcripts encoding TMD-containing secretome proteins contain SECReTE10 (*i*.*e*. ≥1 SECReTE10) in the second position (*NYN*) than transcripts that encode soluble secreted proteins (Fig 2B). However, the fraction of transcripts coding for soluble secreted proteins that contain at least one SECReTE10 in the third position (*NNY*) is even higher. This provides compelling evidence for SECReTE10 enrichment in transcripts that is independent of encoded TMD regions. Correspondingly, when we removed the TMD sequences from mRNAs encoding membrane proteins, we found that these transcripts were no longer enriched with the *NYN*-based form of SECReTE10 (Fig 2C). In contrast, SECReTE10 remained abundant at the third position, *NNY*, after TMD removal (Fig 2C). Thus, TMD sequences contribute to *NYN-*based SECReTE abundance. Finally, we note that the removal of TMD sequences from genes encoding secretome and non-secretome proteins did not alter the overall enrichment of SECReTE in those secretome messages versus non-secretome messages, nor did it change the threshold for differentiating between these groups (S1E Fig).

As contiguous stretches of codons for hydrophobic amino acids (*i*.*e*. TMDs) foster SECReTE abundance, we also examined whether the removal of signal sequence coding regions (SSCRs), which encode signal peptides, from secretome genes had an effect upon the computational analysis of the SECReTE score (Fig 2D). However, the results indicate that no significant change in the overall fraction of secretome genes bearing SECReTE is incurred upon SSCR removal and this is not altered by TMD presence or motif position (compare Fig 2D to 2B). Thus, SSCRs do not contribute extensively to SECReTE abundance.

Since SRP depletion does not block the co-translational ER targeting of mRNAs encoding predicted SRP-independent proteins [24], we examined whether SRP-independent transcripts on yeast ER-bound ribosomes are more enriched with SECReTE10 than SRP-dependent ones (S2A–S2D Fig). Using this dataset [24], we found that both SRP-dependent and -independent transcripts contain SECReTE10 (S2B Fig). However, SRP-dependent (*i*.*e*. TMD-containing) transcripts essentially bear only the *NYN-*based motif, whereas both *NYN-* and *NNY-*based motifs appear in SRP-independent transcripts (S2B and S2C Fig). Moreover, the subset of mRNAs that remain ER-bound after SRP depletion all appear to be enriched in *NNY-*based SECReTE (S2D Fig).

### SECReTE is the principal repetitive sequence motif in secretome transcripts

Repetitive nucleotide triplet sequence elements [*i*.*e*. *NNX*; where *X* = is any of five other dinucleotide combinations: K (T/G), M (C/A), R (A/G), S (G/C), or W (A/T)] other than *NNY* might exist in secretome transcripts and distinguish them from non-secretome transcripts. Thus, we examined for the presence of ≥10 uninterrupted *NNX* repeats in the coding sequences of secretome and non-secretome proteins. Examination of the yeast genome did not reveal any significant repetitive *NNX*-based triplet repeats in mSMPs, except for that seen for pyrimidine (*i*.*e*. *NNY*). Moreover, this did not change upon removal of the TMD sequences from the analysis, indicating that concatenation of the regions flanking the TMD does not create SECReTE motifs *de novo* (S3A and S3B Fig). In contrast, we did find that R (purine) and W repeats are enriched in the third position of a large fraction of non-secretome transcripts, especially after TMD removal (S3A and S3B Fig). This indicates that SECReTE is the principal, if not sole, nucleotide triplet motif of ≥10 repeats in the secretome protein-encoding genes of yeast, although a repetitive *NNR* motif could be identified in the third position of a subset of the non-secretome genes. No additional motifs in either the first or second positions (*i*.*e*. *XNN*, *NXN*) were identified in non-secretome genes.

Next, we examined the distribution of SECReTE in the different regions (*i*.*e*. 5’UTR, CDS, 3’UTR) of yeast genes. We found that the large majority (>90%) of SECReTE motifs (8211 out of 9003) are present in the CDS regions (S4A Fig, left), however, the overall distribution is biased to the 5’ and 3’UTRs, when normalized for the mean length of these smaller regions (S4A Fig, right). Secretome transcripts (1144) contained ~35% of the total SECReTE motifs, an amount proportionally larger than that of non-secretome transcripts (4760), although the motif is more or less evenly represented in both the CDS and separate UTR regions after normalization for length. Mapping of motif distribution along the entire gene length (after normalization) revealed a uniform distribution in TMD-containing transcripts, but also showed that a number of transcripts encoding soluble proteins (~60) have a preference for SECReTE at the 5’ end, as this distribution could be eliminated upon SSCR removal (S4B Fig). Thus, despite the fact the SSCRs do not highly contribute to overall SECReTE abundance (Fig 2D), this does not exclude the possibility that the motif cannot be present therein. Examination of both *NYN-* and *NNY-*based SECReTE motifs in the coding regions showed that both contribute to motif presence at the 5’ end of the same subset of transcripts encoding soluble secreted proteins (S4B Fig).

When comparing SECReTE motifs residing in the CDS to those in the UTRs, we found that CDS motifs tend to consist of *RRY* repeats rather than *NNY-*based repeats (S4C Fig). On the other hand, UTR-residing SECReTE motifs are statistically more pyrimidine-rich and, thus, are biased towards the *NNY* pattern of repeats (S4C Fig). Next, we checked if the UTRs of the secretome transcripts are enriched with pyrimidines in general. Indeed, we found that secretome transcripts have a slightly higher Y content in their UTR (S4D Fig), however, this enrichment disappears after removal of the SECReTE-containing UTRs from the analysis (*e*.*g*. 43 and 99 transcripts for the 5’ and 3’UTRs, respectively) (S4E Fig). This implies that SECReTE motifs contribute to the pyrimidine-enrichment of UTRs in secretome transcripts.

### SECReTE abundance is not dependent upon codon usage

There is a possibility that SECReTE enrichment results from codon usage of the transcript. To check this possibility, we performed permutation test analysis. In this case, each gene sequence was randomly shuffled (1000 times), while codon usage remained constant. We then calculated the Z-score (*i*.*e*. number of standard deviations from the mean) of SECReTE10 for each gene to evaluate the probability of the signal to appear randomly. By looking at Z-score distribution in secretome and non-secretome genes, it can be concluded that SECReTE enrichment in mSMPs is not a random consequence of codon usage (S5A Fig). This conclusion is valid for mSMPs encoding both membrane and soluble proteins (S5B Fig). We also conducted the analysis for each nucleotide position of the codon separately (*i*.*e*. for the *YNN*, *NYN*, and *NNY* versions of the motif). For that, we calculated the fraction of genes with a significant Z-score (≥1.96) for each position separately. The fraction of genes with a significant Z-score was larger in secretome genes than in the non-secretome genes at both the second and third positions of the codon (S5C Fig), strengthening the notion that SECReTE is significantly more enriched in those positions. This finding is not dependent on the presence of TMDs, since the fraction of genes with a significant Z-score was larger for both soluble and TMD-containing secretome transcripts, rather than for soluble and TMD-containing non-secretome transcripts (S5D Fig).

### Cell wall, signal peptide- and TMD-containing yeast proteins in yeast are enriched with SECReTE

**Fig 3:**
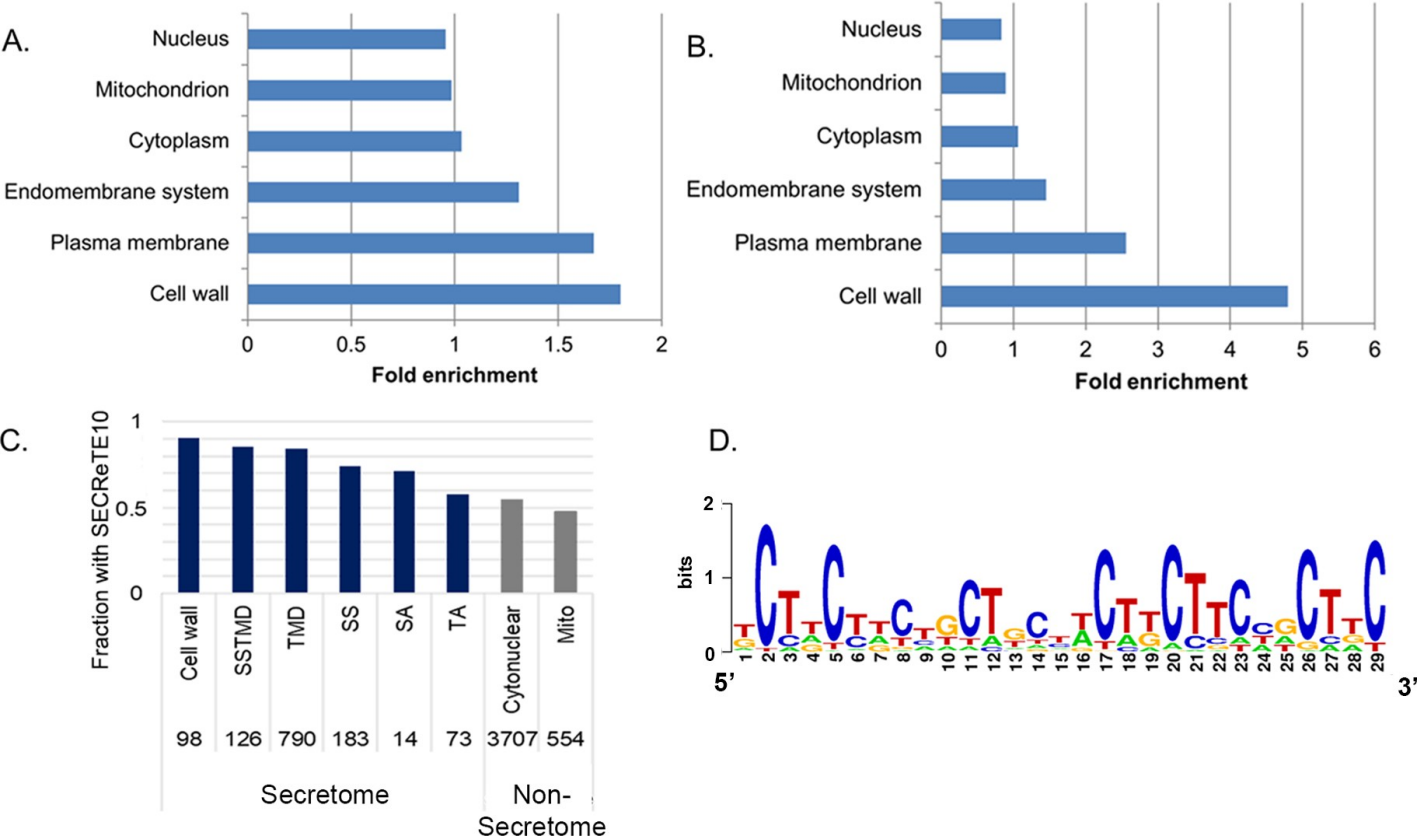
Cell wall proteins are highly enriched with SECReTE. **(A) Cellular component ontology analysis for genes containing SECReTE10.** Genes encoding cell wall proteins, as well as membrane proteins, show the highest and most significant enrichment score. **(B) Cellular component ontology analysis for genes containing SECReTE15.** Genes encoding cell wall proteins are the most enriched with SECReTE. **(C) SECReTE10 abundance in different groups of genes.** More than 90% of mRNAs encoding proteins annotated to localize to the cell wall contain SECReTE. High SECReTE abundance was also noticed in other secretome groups except tail-anchored (TA) proteins. Mitochondrial mRNAs (Mito) have low SECReTE abundance. Numbers above bars represent the number of genes in each group. **(D) MEME analysis of cell wall transcripts**. A motif similar to SECReTE was revealed in cell wall transcripts using MEME. Numbers on the *x* axis indicate base number.

To determine those gene categories that are overrepresented with SECReTE-containing genes, gene ontology (GO) enrichment analysis was conducted. When genes that contain at least one occurrence of SECReTE10 in any of its *YNN-*, *NYN-* or *NNY-*based forms were searched for GO enrichment (using all yeast genes as a background), unsurprisingly, membrane proteins were found to have a high enrichment score (fold enrichment = 1.67) (Fig 3A). The most SECReTE-enriched gene category was that comprising cell wall proteins (fold enrichment = 1.8) (Fig 3A). When 15 *NNY* repeats served as a threshold, the fold-change enrichment of the cell wall protein category increased to 4.8-fold (Fig 3B). To further characterize the mRNAs enriched with SECReTE, we divided the secretome and non-secretome into subgroups and calculated the fraction of transcripts containing SECReTE10 in each category. In agreement with the GO analysis, more than 90% of mRNAs coding for cell wall proteins possess SECReTE10 (and above) motifs and the cell wall proteins were the most SECReTE-rich overall (Fig 3C). Interestingly, this group also comprised the principal set of transcripts that remain associated with ER-bound ribosomes after SRP depletion (S2E and S2F Fig). In addition, we found that 86% of mRNAs of proteins encoding both TMD and signal-sequence (SS) regions, as well as 84% of TMD-encoding secretome mRNAs, contain SECReTE10 (Fig 3C). Of these, mRNAs encoding tail-anchored (TA) proteins contain the lowest number of transcripts with SECReTE10 in the secretome (Fig 3C). TA proteins are known to translocate to the ER through an alternative pathway (GET) after being translated in the cytosol [32–34], and their transcripts are not enriched on ER membranes [22,23] either before or after SRP depletion [24]. This implies that SECReTE is more abundant in mRNAs undergoing translation on the ER. In contrast, transcripts for non-secretome proteins (*i*.*e*. mitochondrial and cytonuclear) have the lowest abundance of SECReTE elements (Fig 3C).

Since SECReTE is highly enriched in mRNAs coding for cell wall proteins, we wanted to check if it could be discovered using an unbiased motif search tool. For that, we analyzed the mRNA sequences of cell wall proteins using MEME to identify enriched mRNA motifs. The most significant result obtained highly resembled the SECReTE10 repeat with either U or C (Fig 3D). Importantly, we did not detect a protein motif within this mRNA motif, eliminating the possibility that the SECReTE element is dependent on a specific protein sequence.

### SECReTE enrichment in secretome transcripts occurs in both prokaryotes and higher eukaryotes

**Fig 4:**
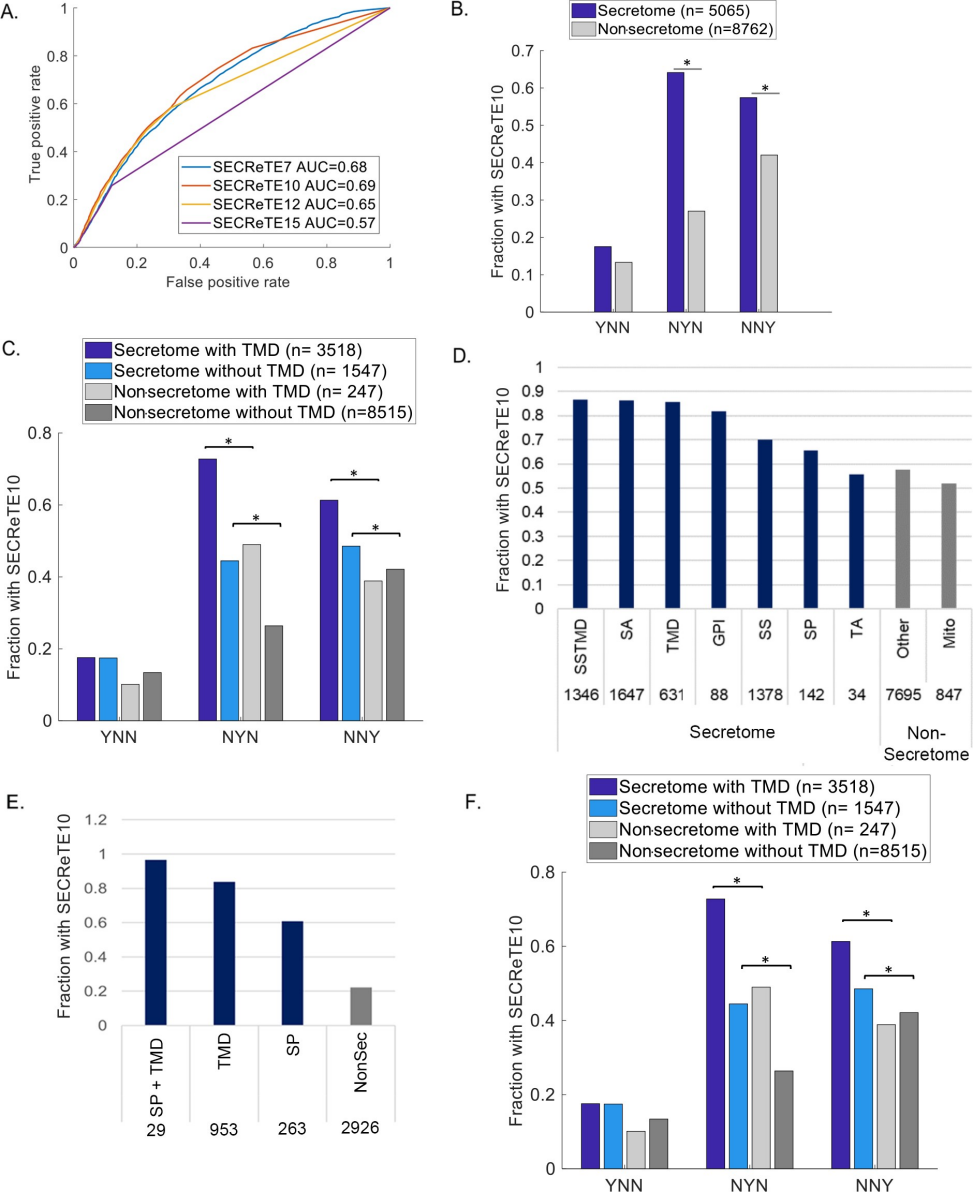
SECReTE enrichment in the secretomes of yeast, bacteria, and humans. **(A) SECReTE10 maximizes the ability to classify secretome genes in human.** ROC curves were plotted for each of the indicated thresholds. Secretome genes were used as the true positive set and non-secretome genes as the true negative set. The AUC (area under the curve) of SECReTE10 was the highest. **B. SECReTE is highly abundant in the mRNAs of human secretome proteins.** SECReTE10 abundance was calculated for each codon position separately. SECReTE abundance in human mSMPs is most significant in the second position of the codon, but highly significant differences were also detected in the third position. *p≤ 3.73E-68, Chi-square test **C. SECReTE is highly abundant in mRNAs coding for soluble secretome proteins in humans.** SECReTE10 presence was examined separately for TMD-containing proteins and soluble secreted proteins. A higher fraction of mRNAs coding for soluble secreted proteins (Secretome without TMD; cyan) contains SECReTE in comparison to non-secretome transcripts without a TMD (light gray). The fraction of soluble secreted proteins having SECReTE in the third position is larger than that of TMD-containing non-secretome proteins (NNY) and is significant. n represent the number of genes in each group. *p ≤ 3.49E-12, Chi-square test. (**D) SECReTE10 abundance in different groups of human genes.** High SECReTE abundance was observed for other secretome protein groups, except tail-anchored (TA) proteins. Mitochondrial mRNAs (Mito) have low SECReTE abundance. Numbers above bars represent the number of genes in each group. **(E) SECReTE10 abundance in *B*. *subtilis*.** SECReTE10 abundance was scored and was observed to be higher in mRNA coding for genes encoding secretome proteins (*i*.*e*. SS&TMD, TMD, and SS) as compared to those encoding non-secretome (Non-Sec) proteins. Numbers under bars represent the number of genes in each group. **(F) SECReTE10 abundance in *S*. *pombe*** SECReTE10 abundance was calculated for each codon position separately for TMD-containing proteins and soluble secreted proteins. A higher fraction of mRNAs coding for soluble secreted proteins (Secretome without TMD; cyan) contains SECReTE in comparison to non-secretome transcripts, either with or without a TMD (Non-secretome with TMD; dark gray, Non-secretome without TMD; light gray). The fraction of soluble secreted proteins having SECReTE in the third position is larger than that of TMD-containing non-secretome proteins (NNY) and is significant. n represent the number of genes in each group. n represent the number of genes in each group. * *p*≤ 5.63E-3, Chi-square test.

Conservation or convergence in evolution are often strong indications of functional significance. To check whether SECReTE enrichment in mSMPs is found in additional organisms (*e*.*g*. humans and *B*. *subtilis*) we analyzed other genomes. In humans, as in *S*. *cerevisiae*, SECReTE10 gave the most significant separation between RNAs encoding secretome and non-secretome proteins, based on ROC analysis (Fig 4A). After verifying that SECReTE10 does not correlate with gene length, SECReTE10 served as a threshold to define presence of the SECReTE motif. As in yeast, SECReTE is enriched in the second and third codon positions of secretome transcripts, in comparison to non-secretome transcripts (Fig 4B). Also, a larger fraction of secretome transcripts that lack TMDs contain the *NNY-*based SECReTE, as compared to non-secretome transcripts bearing TMDs (Fig 4C). Thus, the SECReTE motif is present in higher organisms. A list of all SECReTE10 and higher motifs found in human genes is given in S4 Table. Interestingly, unlike yeast, we found that a disproportionally large majority of motifs (29753 out of 52,047) are present in the UTRs instead of being in the CDS, especially after normalization for length, and this phenomenon is observed for both secretome and non-secretome transcripts (S6A Fig). Therefore, in contrast to yeast, the UTRs and especially the 3’UTR are preferential sites for SECReTE location in human transcripts. Like yeast, however, *RRY* enrichment is observed for SECReTE motifs in the CDS regions, while high pyrimidine content is observed in the UTRs (S6B Fig). Therefore, although both yeast and human share the same SECReTE motifs, their distribution over gene region appears different. Interestingly, human transcripts encoding glycophosphatidylinositol (GPI)-anchored proteins, which are equivalent to cell wall proteins, were found to be highly enriched with SECReTE. In fact, a SECReTE-like motif was previously shown to confer the translation-independent localization of a transcript encoding human GPI-anchored protein, placental alkaline phosphatase, to the ER [35] In contrast, tail-anchored genes, as well as mitochondrial and cytonuclear genes, have a low SECReTE abundance as seen in yeast (Fig 4D). Finally, we also detected a high abundance of SECReTE10 in genes encoding secretome proteins from *B*. *subtilis*, in comparison to those encoding non-secretome proteins (Fig 4E). Thus, SECReTE motifs are also present in prokaryotic genomes.

We next asked whether SECReTE is conserved evolutionarily via inheritance. To differentiate between conservation and possible convergence we analyzed the genome of the fission yeast, *S*. *pombe*, for the presence and position of SECReTE in secretome and non-secretome transcripts. As found for *S*. *cerevisiae*, SECReTE is enriched (in both *NYN-* and *NNY-*based forms) in a larger fraction of *S*. *pombe* mSMPs that lack TMDs, as compared those containing TMDs or to non-secretome transcripts that either bear or lack TMDs (Fig 4F). Next, we aligned orthologous genes encoding secretome proteins from *S*. *cerevisiae* to those of *S*. *pombe* (457 genes total), and examined whether SECReTE is found in the same (*i*.*e*. aligned) position within the gene. We found that the coordinates of SECReTE motifs in the large majority (*e*.*g*. 393 out of 457) of ortholog pairs were non-aligned. This might imply that the majority of SECReTE motifs arose through convergent evolution, although we cannot rule out drift of the motif after species divergence. Nonetheless, it is clear that SECReTE is present in all species examined by us, from prokarya to eukarya, the latter including yeasts and mammals.

### Design of mutations in SECReTE to examine the effects upon protein secretion

To further understand the significance of SECReTE and validate its importance to yeast cell physiology, we examined its relevance by elevating or decreasing the signal in selected genes. Three representative genes were chosen, based on their relatively short gene length, a detectable phenotype upon their deletion, and their function in different physiological pathways. These genes included: *SUC2*, which encodes a soluble secreted periplasmic enzyme; *HSP150*, which encodes a soluble media protein; and *CCW12*, which encodes a GPI-anchored cell wall protein. The overall SECReTE signal of the genes was increased by substituting any A or G found in the third codon position with a T or C, respectively, thereby enriching SECReTE presence along the entire gene [(+)SECReTE]. The reverse substitution, converting T to A or C to G, decreased the overall SECReTE signal [(-)SECReTE]. We note that we added or removed only *NNY-*based triplet motifs, in order not to change the amino acid sequence of the encoded protein. The number of motifs present in each gene before and after SECReTE addition/reduction is shown in S5 Table. Thus, in the case of *HSP150* several *NYN-*based SECReTE motifs remain in the (-)SECReTE mutant. Changes in the stability of the mRNA secondary structure (free energy) and the Codon Adaptation Index (CAI) [32] were kept to within a similar range (S5 Table). SECReTE mutations in *SUC2*, *HSP150*, and *CCW12* are shown along the length of the gene, using a minimum threshold of either 1 *NNY* repeats or 10 *NNY* repeats, as shown in S7 Fig (S7A–S7C Fig; upper and lower parts, respectively).

### SECReTE mutations in *SUC2* alter invertase secretion

**Fig 5:**
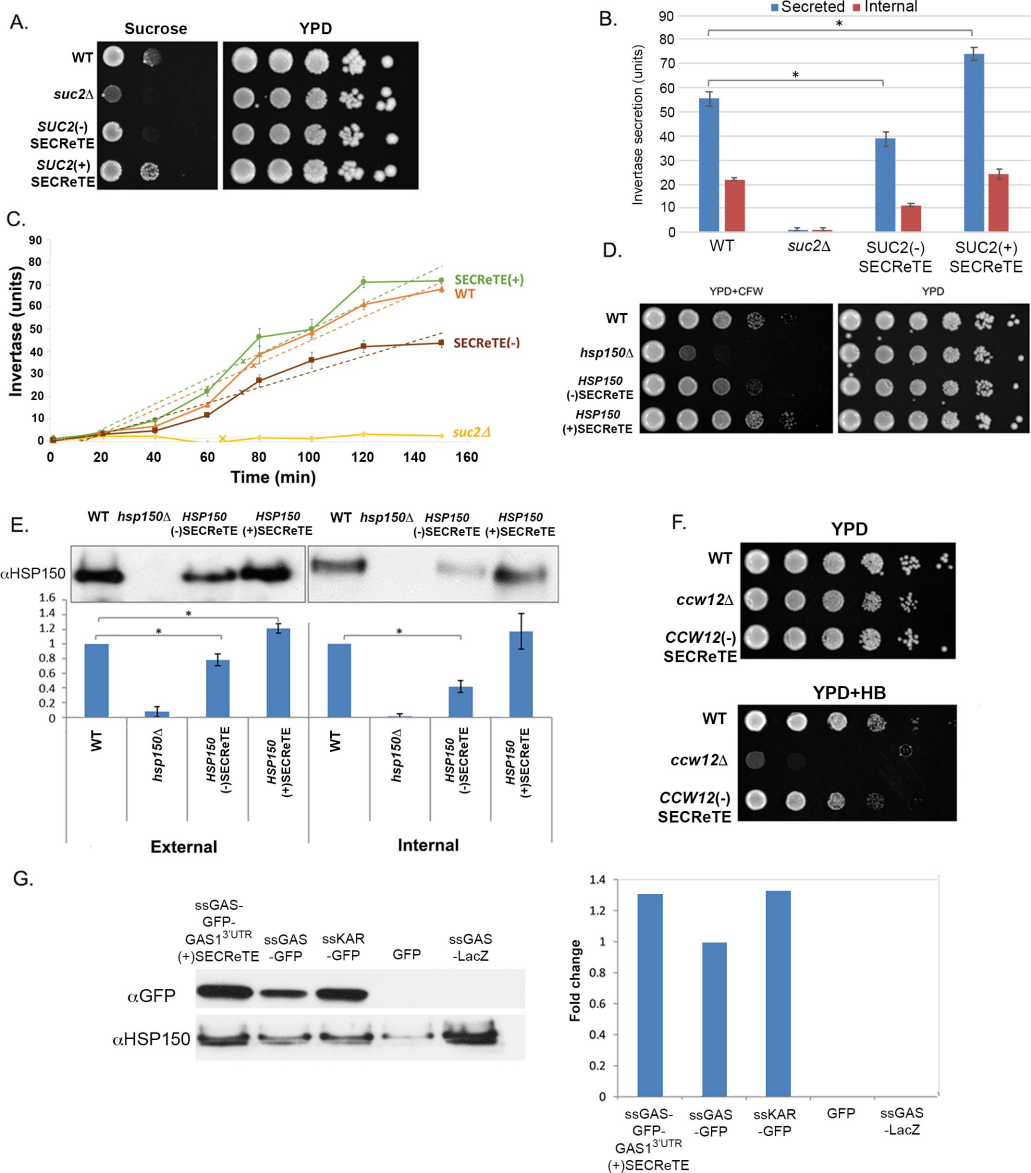
The levels of secretion of endogenous and exogenous proteins are affected by SECReTE strength. **(A) SECReTE enhances the ability to grow on sucrose.** The ability of WT, *suc2Δ*, *SUC2*(+)SECReTE and *SUC2*(-)SECReTE yeast to grow on sucrose was examined by drop-test. Cells were grown to mid-log on glucose-containing YPD medium, prior to serial dilution and plating onto sucrose-containing synthetic medium or YPD. Cells were grown for 2 days prior to photo-documentation. The *SUC2*(-)SECReTE mutant exhibited reduced growth than WT cells, while *SUC2*(+)SECReTE cells exhibited better growth. *suc2Δ* cells were unable to grow on sucrose-containing medium. (**B) SECReTE enhances invertase secretion.** The indicated strains from *A* were analyzed using an invertase secretion assay. Both internal and secreted invertase activity was measured in units (1 U = 1 μmol glucose released/min per O.D._600_ unit) after glucose de-repression. Both activities were reduced in *SUC2*(-)SECReTE cells and elevated in *SUC2*(+)SECReTE cells. Error bars represent the standard deviation from three experimental repeats. *p<0.0161 (t-test). **(C) SECReTE presence enhances the rate of invertase secretion.** The indicated strains from *A* were incubated in low glucose medium for varying times (0-150min) and analyzed using the invertase secretion assay, as in *B*. Secreted invertase activity was measured in units (1 U = 1 μmol glucose released/min per O.D._600_ unit) after glucose de-repression. Error bars represent the standard deviation from three experimental repeats. Linear regression of the data (dotted lines) from inverse reciprocal plots was used to determine the T_1/2_ for half-maximal accumulation and rate; “x” mark on line indicates time of half-maximal accumulation. The rate of invertase secretion is enhanced in (+)SECReTE cells and reduced in (-)SECReTE cells relative to WT. **(D) SECReTE enhances the ability to grow on calcofluor white.** The ability of WT, *hsp150Δ*, *HSP150*(+)SECReTE and *HSP150*(-)SECReTE cells to grow on CFW was examined by drop-test. Cells were grown to mid-log on YPD, prior to serial dilution and plating on YPD alone or YPD plates containing CFW, and incubated at 30°C. Cells were grown for 2 days prior to photodocumentation. The *HSP150*(-)SECReTE mutant exhibited hypersensitivity in comparison to WT cells, while *HSP150*(+)SECReTE cells were less sensitive. *hsp150Δ* cells grew poorly on medium containing CFW. **(E) SECReTE enhances Hsp150 secretion.** The indicated strains from *D* were subjected to the Hsp150 secretion assay. Cells were grown to mid-log phase at 37°C for 4hrs and examination in cell lysates (internal) or medium (external) by Western analysis using anti-Hsp150 antibodies. External Hsp150 was decreased in *HSP150*(-)SECReTE cells in comparison to WT, while it was increased in the *HSP150*(+)SECReTE strain. Internal Hsp150 was decreased in *HSP150*(-)SECReTE cells and also slightly in *HSP150*(+)SECReTE cells, in comparison with WT cells. No internal nor external Hsp150 was detected in the lysate or medium derived from *hsp150Δ* cells, respectively. Band intensity was quantified using ImageJ and presented in the histogram below. A representative experiment is shown in the top panels for both external and internal Hsp150 secretion. The graphs below represent the ratio of the intensity of all samples relative to that of WT for three biological repeats, *p<0.05 (t-test). Error bars represent the standard deviation. **(F) SECReTE enhances the ability to grow on hygromycin B.** The ability of WT, *ccw12Δ*, and *CCW12*(-)SECReTE cells to grow on HB was examined by drop-test. Cells were grown to mid-log on glucose-containing YPD medium, prior to serial dilution and plating onto HB-containing YPD or YPD alone. Cells were grown for 2 days prior to photodocumentation. The *CCW12*(-)SECReTE strain was more sensitive to HB stress in comparison to WT cells. *ccw12Δ* cells were unable to grow on medium containing HB. (**G) SECReTE enhances secretion of an exogenous protein, SSGAS1-GFP.** Yeast expressing SSGAS1-GFP-3’GAS1^UTR^(+)SECReTE, SSGAS1-GFP, SSKAR2-GFP, GFP, and SSGAS1-LacZ from plasmids were grown to mid-log phase on synthetic medium containing 2% raffinose and shifted to 3% galactose-containing medium for 4hrs. Proteins expressed from the different strains were TCA precipitated from the medium and the precipitates resolved by SDS-PAGE. GFP was detected with an anti-GFP antibody, while Hsp150 was detected with an anti-Hsp150 antibody and was used as a loading control. Band intensity was quantified using ImageJ; intensity was scored relative to SSGAS1-GFP secretion. Addition of the *GAS1* 3’UTR mutated to contain SECReTE improved the secretion of SS-Gas1 and was comparable to that of SSKAR2-GFP. GFP lacking a signal sequence (GFP) was not secreted and SSGAS1-LacZ was used as a negative control for GFP detection.

*SUC2* codes for different forms of invertase translated from two distinct mRNAs, short and long, which differ only at their 5’ ends. While the longer mRNA codes for a secreted protein that contains a signal sequence, the signal sequence is omitted from the short isoform, which codes for a cytoplasmic protein. Secreted Suc2 expression is subjected to glucose repression; however, under inducing conditions (*i*.*e*., glucose depletion), Suc2 is trafficked through the secretory pathway to the periplasmic space of the cell. There, it catalyzes the hydrolysis of sucrose to glucose and fructose, this enzymatic activity being responsible for the ability of yeast to utilize sucrose as a carbon source and can be measured by a biochemical assay (*i*.*e*. invertase activity), both inside and outside of the cell. The effect of SECReTE mutations on Suc2 function was tested by examining the ability of mutants to grow on sucrose-containing media by drop-test. Interestingly, the growth rate of *SUC2*(-)SECReTE on sucrose plates was decreased, while the *SUC2*(+)SECReTE mutant exhibited better growth in comparison to WT cells (Fig 5A), even though no growth change was detected on YPD plates. These findings suggest that SECReTE strength affects the secretion of Suc2. These changes in Suc2 secretion could result from changes in *SUC2* transcription, Suc2 production, and/or altered rates of secretion. To distinguish between possibilities, WT cells, *suc2Δ*, and *SUC2* SECReTE mutants were subjected to invertase assays. The invertase assay enables the quantification of secreted Suc2, as well as internal Suc2, by calculating the amount of glucose produced from sucrose. As expected, under glucose repressing conditions (*e*.*g*. 2% glucose) the levels of both secreted and internal Suc2 were very low. When cells were grown on media containing low glucose (*e*.*g*. 0.05% glucose) to promote the expression of the secreted enzyme, secreted Suc2 levels were altered due to changes in SECReTE. Corresponding to the drop-test results (Fig 5A), a significant decrease in secreted invertase was detected with *SUC2*(-)SECReTE cells, while a significant increase was detected with *SUC2*(+)SECReTE cells, in comparison to WT cells (Fig 5B). Importantly, *SUC2*(+)SECReTE cells were found to secrete nearly 2-fold (92.2±9.2%, *p <*0.016) more invertase than *SUC2*(-)SECReTE cells, while no Suc2 secretion was detected from *suc2Δ* cells (Fig 5B, secreted). If SECReTE mutations affect the efficiency of Suc2 secretion, but not its synthesis, then Suc2 should accumulate in *SUC2*(-)SECReTE cells corresponding to the difference secreted from *SUC2*(+)SECReTE cells. However, this was not the case as the internal amount of Suc2 decreased in *SUC2*(-)SECReTE cells and slightly increased in *SUC2*(+)SECReTE cells (Fig 5B, internal). These findings suggest that SECReTE alterations in *SUC2* might affect the level of protein production. We next examined the rate of invertase secretion for WT, *SUC2*(+)SECReTE, and *SUC2*(-)SECReTE cells shifted to low glucose medium for varying amounts of time (Fig 5C). The results show that the average maximal rate of secretion from *SUC2*(+)SECReTE cells is slightly higher than for WT cells (*i*.*e*. 0.479±0.016 vs. 0.432±0.013 units per min per O.D._600_ unit of cells; ±standard deviation, n = 3 experiments), and was significantly (62.4±7.8%; *p <*0.0001) higher than of *SUC2*(-)SECReTE cells (0.295±0.022 units/min per O.D._600_ unit of cells; ±standard deviation, n = 3 experiments). In contrast, the time required to achieve half-maximal secretion between *SUC2*(+)SECReTE and *SUC2*(-)SECReTE cells was relatively unchanged under the experimental conditions (*i*.*e*. ~74 min; R^2^ values = >94). Thus, the presence of SECReTE affects not only invertase production and overall secretion, but also its rate of secretion from yeast.

### SECReTE mutations alter Hsp150 secretion and cell wall stability

Next, we wanted to study the importance of SECReTE in *HSP150*. Hsp150 is a component of the outer cell wall and while the exact function of Hsp150 is unknown, it is required for cell wall stability and resistance to cell wall-perturbing agents, such as Calcofluor White (CFW) and Congo Red (CR). While *hsp150Δ* cells are more sensitive to cell wall stress, the overproduction of Hsp150 increases cell wall integrity [36]. Hsp150 is secreted efficiently into the growth media and its expression is increased upon heat shock [37,38]. The effect of modifying SECReTE in *HSP150* was examined via drop-test by testing the sensitivity of *HSP150*(-)SECReTE and *HSP150*(+)SECReTE cells to added CFW, in comparison to WT and *hsp150Δ* cells. As can be seen from Fig 5D while the *HSP150*(-)SECReTE strain was more sensitive to CFW as compared to WT cells, the *HSP150*(+)SECReTE strain was more resistant to CFW. As expected, *hsp150Δ* cells are the most susceptible to CFW (Fig 5D). *HSP150* strains were also subjected to Western blot analysis to measure levels of the mutant proteins. Since *HSP150* secretion is elevated upon heat-shock [37,38], cells were shifted to 37°C before protein extraction. Protein was extracted from both the growth medium and cells to detect both external and internal protein levels, respectively. The amount of Hsp150 secreted to the medium was decreased in *HSP150*(-)SECReTE cells and elevated in *HSP150*(+)SECReTE cells, in comparison to WT cells (Fig 5E). Similar to Suc2, the internal amount of Hsp150 was decreased in *HSP150*(-)SECReTE cells, relative to WT cells, and showed a greater reduction than that seen in the external form (Fig 5E), despite the fact that several *NYN-*based motifs remain in the gene. As the internal level of Hsp150 in *HSP150*(+)SECReTE cells was more or less unchanged relative to WT cells, we concluded that SECReTE alteration in *HSP150* may also affect protein production.

### SECReTE mutations in *CCW12* alter cell wall stability

*CCW12* encodes a GPI-anchored cell wall protein that localizes to regions of the newly synthesized cell wall and maintains wall stability during bud emergence and shmoo formation. Deletion of *CCW12* was shown to cause hypersensitivity to cell wall destabilizing agents, like hygromycin B (HB) [39,40]. Since the SECReTE score is very high in *CCW12*, it was not possible to further increase the signal. Therefore, we generated only *CCW12*(-)SECReTE cells and tested their ability to grow on HB-containing plates. As seen with *HSP150*(-)SECReTE (Fig 5D), we found that the *CCW12*(-)SECReTE mutation rendered cells sensitive to cell wall perturbation, in comparison to WT cells (Fig 5F).

### SECReTE addition affects secretion of an exogenous naïve protein

The ability of SECReTE addition to improve the secretion of an exogenous protein would not only be substantial evidence for its importance, but also could be a useful, low-cost, industrial tool to improve the secretion of recombinant proteins without changing protein sequence. To test that, we employed a *GFP* transcript construct bearing the encoded signal sequence (SS) of Gas1 (*SSGAS*; SSGas1) at the 5’ end. SSGas1 addition enables the secretion of GFP protein to the medium, although its secretion was not as efficient in comparison to other SS-fused GFP proteins, such as SSKar2 (Fig 5G). To potentially improve the secretion of *SSGAS-GFP*, we added an altered 3’UTR sequence of *GAS1* that contained SECReTE [*i*.*e*. in which all A’s and G’s were replaced with T’s and C’s, respectively; SSGAS-GFP-GAS1^3’UTR^(+)SECReTE]. We then tested the effect of SECReTE addition upon the secretion of GFP into the media. We found that the addition of SECReTE to the 3’UTR of *SSGAS*-*GFP* improved the secretion of GFP secretion into the media, in comparison to SSGAS-GFP, and was similar to that of SSKar2-GFP construct (Fig 5G). GFP expression without the signal sequence was unable to be secreted (Fig 5G).

### The effect of SECReTE mutations on mRNA levels

As protein levels may be altered by (-)SECReTE and (+)SECReTE mutations (Fig 5B, 5E and 5G), we examined whether changes in gene transcription or mRNA stability are involved. Quantitative real-time (qRT) PCR was employed to check whether mRNA levels of *SUC2*, *HSP150*, and *CCW12* are affected by SECReTE strength. We found that *SUC2*(–)SECReTE mRNA levels were almost 30% lower than in *SUC2* WT cells, while *SUC2*(+)SECReTE levels were ~50% higher than WT (S8A Fig). This change in mRNA levels might contribute to the ability of *SUC2*(+)SECReTE mutant to increase protein production and, therefore, grow better on sucrose-containing medium (Fig 5A–5C).

The effect of SECReTE mutation on *HSP150* mRNA levels was also studied. We found that the mRNA level of *HSP150*(-)SECReTE was similar to WT, while that of *HSP150*(+)SECReTE was slightly decreased (S8B Fig). Thus, the change in Hsp150 protein levels and sensitivity to CFW due to SECReTE alteration (Fig 5D and 5E) is not entirely explained by changes in mRNA levels. SECReTE mutations in *CCW12*(-)SECReTE did not cause a significant change in its mRNA level (S8C Fig), therefore, the increased sensitivity of *CCW12*(-)SECReTE to HB (Fig 5F) is probably not due to a decrease in *CCW12* mRNA.

### The addition of SECReTE motifs increases mRNA localization to the ER

To test whether SECReTE has a role in dictating mRNA localization, we visualized the *SUC2* and *HSP150* mRNAs by single-molecule FISH (smFISH) using specific fluorescent probes and tested the influence of SECReTE alteration on the level of mRNA co-localization with the ER. We used Sec63-GFP as an ER marker and calculated the percentage of mRNA granules (spots) per cell that co-localized with cortical and perinuclear ER (cER and nER, respectively) or were not localized to the ER. We note that probes to the native gene sequences were used to measure the level of mRNA localization in WT cells as well as in (+)SECReTE or (-)SECReTE cells. The number of FISH spots per cell was variable for both mRNAs, with (-)SECReTE cells having less spots than WT cells, while (+)SECReTE cells had more spots per cell than WT cells (S9A Fig). This largely reflects the results obtained by qRT-PCR for *SUC2* (S8A Fig), however, we cannot discount the possibility that SECReTE alterations lessen the level of mRNA hybridization with the probe set and, thus, underestimate RNA localization to some degree.

**Fig 6:**
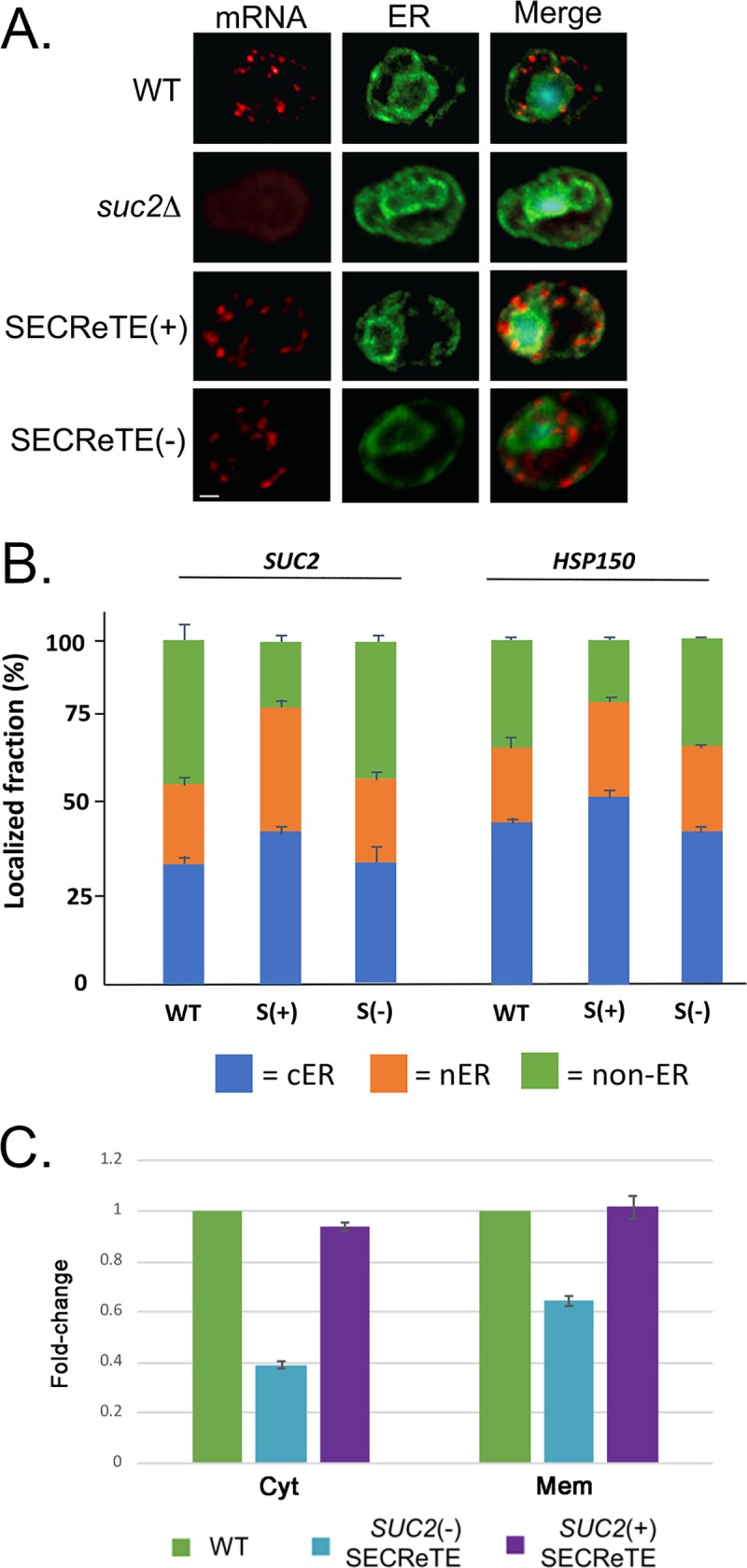
SECReTE abundance enhances mRNA localization to the ER. (**A) Visualization of endogenously expressed *SUC2*(+)SECReTE and *SUC2*(-)SECReTE mRNAs using smFISH**. Yeast endogenously expressing WT *SUC2*, *SUC2*(+)SECReTE, or *SUC2*(-)SECReTE and Sec63-GFP from a plasmid were grown to mid-log phase on SC medium containing 2% glucose prior to shifting cells to low glucose-containing medium (0.05% glucose) to induce *SUC2* expression. Cells were processed for smFISH labeling using non-overlapping, TAMRA-labeled, FISH probes complementary to *SUC2*, prior to labeling with DAPI (shown in *merge*). Representative images are shown. Line = 1μm. **B. Quantification of *SUC2* and *HSP150* (+)SECReTE and (-)SECReTE mRNA localization to the ER.** mRNA granule scoring was performed using the FISH-quant algorithm and co-localization of the granules to the ER (both cER and nER) was performed (see Materials and Methods). The percentage of granules that either co-localized or did not co-localize with Sec63-GFP-labeled cER or nER was scored for each cell. The histogram shows the average score for at least ~50 cells and ~250 granules for each strain examined. *SUC2 p* values: (+)SECReTE vs. (-)SECReTE, *p* < 3.1E-8; WT vs. (+)SECReTE, *p* < 8.1E-10; *HSP150* (+)SECReTE vs. (-)SECReTE, *p* < 0.008 (t-test). **C. Analysis of *SUC2* mRNA localization using cell fractionation and qRT-PCR.** WT *SUC2*, *SUC2*(+)SECReTE, or *SUC2*(-)SECReTE yeast cells were fractionated to membrane and cytosol fractions. RNA was extracted from both fractions and subjected to qRT-PCR analysis to quantify the levels of *SUC2* mRNA in each fraction. Primers were used to amplify the long transcript of *SUC2*, which encodes the secreted protein. Primers for actin were used for normalization. *SUC2*(-)SECReTE cells exhibited lower *SUC2* mRNA levels in both fractions than WT and *SUC2*(+)SECReTE, indicating a loss in stability as well as localization. Error bars represent the standard deviation of three biological repeats.

We found that the level of co-localization between *SUC2*(-)SECReTE mRNA granules and Sec63-GFP was significantly less in comparison to *SUC2*(+)SECReTE mRNA granules (*e*.*g*. 56.7±1.5% vs. 74.1±1.7% co-localization, respectively; *p =* 2.0E-13) (Figs 6A, 6B and S9B), while being similar to native *SUC2* (*e*.*g*. 56.3±2.4% ER co-localization). This finding suggests that the number of SECReTE motifs influences mRNA localization to the ER, in addition to enhancing secretion. We also found that there were fewer granules present in *SUC2*(-)SECReTE cells than observed in either *SUC2*(+)SECReTE or WT cells (S9A Fig), which corresponds with the qRT-PCR results (S8A Fig). Finally, we note that no specific ER subdomain (*i*.*e*. cER or nER) was preferentially labeled upon the increase in SECReTE motifs (Fig 6B).

We next examined the level of *HSP150* mRNA localization to the ER in *HSP150*(+)SECReTE or *HSP150*(-)SECReTE cells. We found that as with *SUC2*, the addition of SECReTE motifs increased the level of ER localization (Fig 6B and S9C Fig) from 63.7±2.0% to 77.9±1.6% over native *HSP150* localization (*p =* 7.0E-8). In contrast, no change in the level of *HSP150*(-)SECReTE mRNA co-localization was observed (*e*.*g*. 64.0±1.6%), which perhaps reflects presence of the *NYN-*based motifs that could not be mutated without altering the amino acid sequence. Overall, however, both sets of results show that SECReTE addition to an mRNA increases the pattern of ER localization. To substantiate the smFISH results for *SUC2*, we also performed the subcellular fractionation of cells expressing native *SUC2*, *SUC2*(-)SECReTE, or *SUC2*(+)SECReTE to obtain crude membrane (containing ER) and cytosolic fractions and quantified the distribution of mRNA using qRT-PCR (Fig 6C). After normalization using actin mRNA as a control, the results indicated that *SUC2*(-)SECReTE mRNA is less abundant overall (as observed above in S8A Fig and S9A Fig) and appeared to be less membrane-associated than either native or *SUC2*(+)SECReTE mRNA by ~40%. Taken altogether, our results imply that SECReTE presence/addition stabilizes secretome mRNAs, increases mRNA localization to the ER, and enhances both protein production and secretion.

### Identification of potential SECReTE-binding proteins

**Fig 7:**
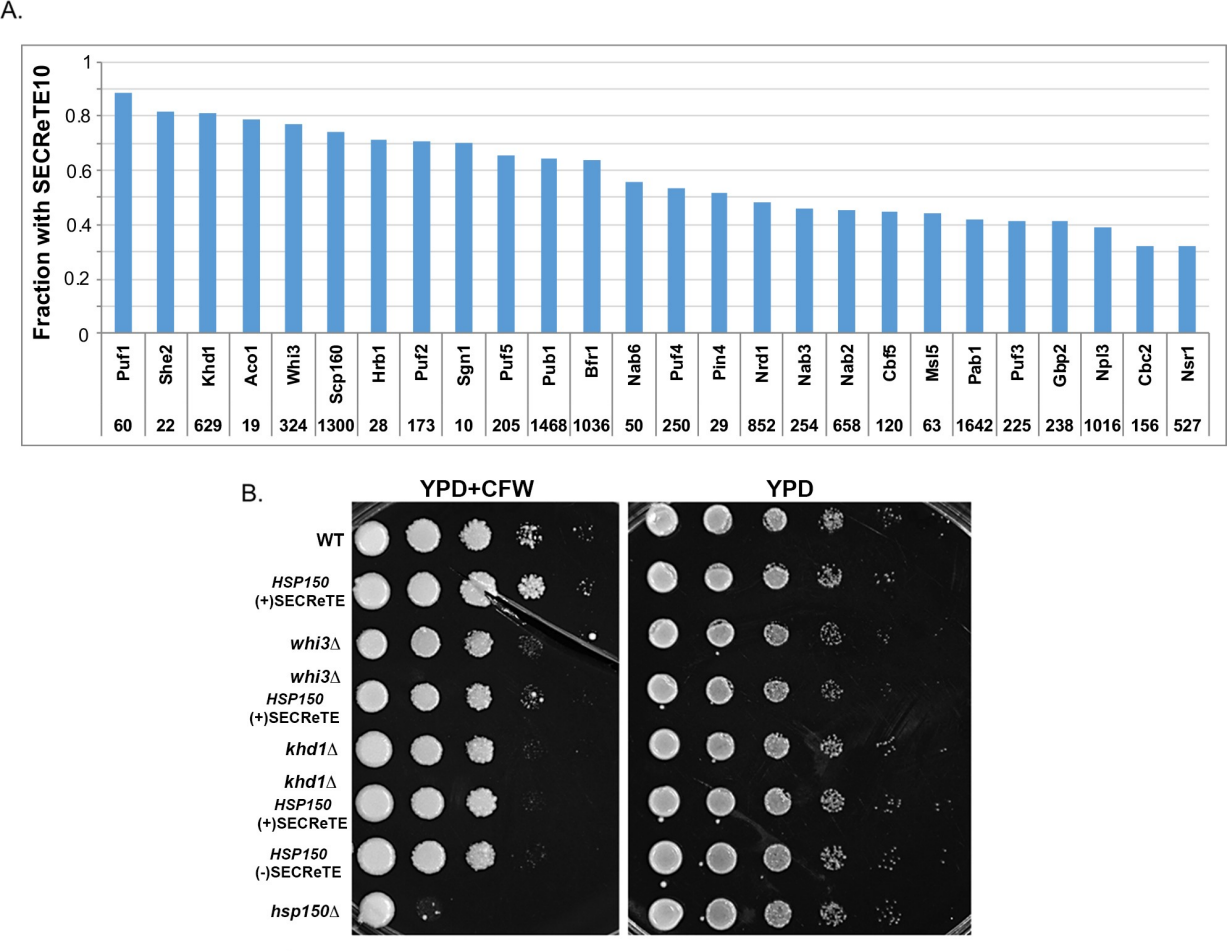
Identification of potential SECReTE-binding proteins. **(A) Identification of SECReTE10-containing transcripts in RNA-binding protein pulldown studies.** The number and fraction of SECReTE10-containing mRNAs from the total mRNAs bound to the indicated RBPs is shown. The microarray analysis data used to generate the histogram was published in references [41–43]. **(B) Identification of potential SECReTE-binding partners**. WT cells and either WT or *HSP150*(+)SECReTE cells deleted for genes encoding the indicated RBPs (*e*.*g*. Whi3, and Khd1) were grown to mid-log phase on YPD at 30°C, prior to serial dilution and plating onto either solid YPD medium or YPD containing CFW. Yeast were grown 2 days prior to photodocumentation.

To further elucidate the role of SECReTE it is essential to identify its binding partners, presumably RBPs. Large-scale approaches were previously used to identify mRNAs that are bound >40 known RBPs in yeast [41–43]. To obtain a list of potential SECReTE-binding proteins (SBPs) we searched the datasets for RBPs that bind mRNAs highly enriched with SECReTE. For each RBP, we calculated what fraction of its bound transcripts contain SECReTE10. RBPs found to bind large fractions of SECReTE10-containing mRNAs included Bfr1, Whi3, Puf1, Puf2, Scp160, and Khd1 (Fig 7A), and were all previously shown to bind mSMPs [41–43]. To test which of these candidates bind SECReTE, each of the genes these RBPs was deleted in either WT or *HSP150*(+)SECReTE cells. We hypothesized that the deletion of a genuine SBP might confer hypersensitivity to CFW and eliminate the growth rate differences between WT and *HSP150*(+)SECReTE cells observed on CFW-containing plates (Fig 5D). When *PUF1*, *PUF2*, or *SHE2* were deleted we found that *HSP150*(+)SECReTE strain was still more resistant to CFW than WT cells (S10 Fig). One possible explanation for this lack of effect is that these RBPs either do not bind *HSP150* or that they are redundant with other SBPs. However, we did find that the deletion of either *WHI3* or *KHD1* eliminated the differences between WT and *HSP150*(+)SECReTE strains on CFW-containing plates (Fig 7B). This suggests Whi3 and Khd1 bind *HSP150* mRNA and possibly other secretome mRNAs, and even WT cells alone were rendered more sensitive to CFW in their absence (Fig 7B).

## Discussion

The correct sorting of proteins within the cell is crucial for cellular organization and normal function. While the information for protein localization can reside within the protein sequence (*e*.*g*. protein targeting sequences), the spatial localization of an mRNA may also be important for protein proper targeting cell [1,2]. For example, mSMPs localize to the surface of the ER independently of translation and that localization requires elements within the transcript that are presumably recognized by an ER-localized RBP (see reviews [8,9,44]). It was shown previously that ER-targeted TMD-containing proteins are highly enriched with amino acids containing uracil-rich codons [31] and, thus, their ORFs are enriched with pyrimidines [27]. Nevertheless, mRNAs coding for secretome proteins that do not contain TMDs were also found to be enriched on ER membranes [2,14,45]. Therefore, an additional mechanism or element appears necessary to confer mSMP localization. Here, we identify features that characterize all mSMPs, either encoding a TMD or not, and discovered a repetitive motif consisting of ≥10 consecutive *NNY* repeats. This motif, termed SECReTE, is not restricted to transcripts coding for TMD-containing proteins, but can be found in higher abundance in all secretome transcripts, from prokaryotes (*e*.*g*. *B*. *subtilis*) to yeast (*S*. *cerevisiae* and *S*. *pombe*) to humans (Figs 1 and 4). By analyzing the *S*. *pombe* genome it was discovered that SECReTE tends to be positioned differently than in orthologous *S*. *cerevisiae* genes encoding secretome proteins. This implies that SECReTE enrichment in mSMPs may have evolved in a number of different ways (*e*.*g*. conservation, drift, or convergence). Correspondingly, we found that SECReTE is preferentially located in the 3’UTR of human transcripts, while being present mainly in the CDS of budding yeast (S4A and S6A Figs). The idea that SECReTE motifs are present throughout evolution likely emphasizes its significance and functionality.

To better characterize SECReTE, we first determined the number of *NNY* repeats that can serve as a threshold to verify its presence and found that ten (*i*.*e*. SECReTE10) constitute a genuine motif, rather than a random occurrence, and enabled significant separation between secretome and non-secretome mRNAs (Fig 1). Importantly, no other repetitive motif was identified in secretome transcripts (S3A and S3B Fig). SECReTE abundance was calculated separately for each position of the codon and while being barely present in the first position (Fig 2A, *YNN*), it was highly represented in the second and third positions in mSMPs (*NYN* and *NNY*, respectively), in comparison to non-mSMPs. Interestingly, the SECReTE10-containing fraction of transcripts coding for soluble secreted proteins is larger than that of mRNAs encoding secreted membrane proteins, suggesting that SECReTE enrichment is not merely due to the high fraction of TMD-containing genes in the secretome (Fig 2B). Importantly, when encoded TMD sequences were removed from the analysis, SECReTE10 was found to be more abundant in the third position of the codon (*NNY*) in secretome transcripts (Fig 2C). In contrast, no significant change in SECReTE abundance was observed upon removal of the SSCR regions from the computational analysis of secretome genes that encode signal peptides (Fig 2D). Thus, it is the TMD regions that contribute to *NYN-*based SECReTE motif enrichment.

By analyzing the ribosome profiling datasets of both Jan *et al*. [22] and Chartron *et al* [23], we verified that a higher fraction of SECReTE10-containing transcripts is enriched on ER-bound ribosomes (S1A Fig) and in polysomes extracted from the membrane fraction (S1B Fig), as well as in the membrane fraction itself (S1C Fig). In contrast, transcripts with SECReTE10 were not enriched on mitochondria-bound ribosomes (S1D Fig). Moreover, analysis of a recent dataset by Costa *et al* [24] revealed that conditional SRP depletion strongly affects the association of predicted SRP-dependent (*i*.*e*. TMD-containing) transcripts with ER-bound ribosomes, but had less effect upon SRP-independent transcripts that are more enriched with *NNY-*based SECReTE motifs (S2 Fig). Permutation analysis confirmed that SECReTE enrichment in mSMPs is not arbitrary and demonstrated that it is independent of codon composition (S5 Fig). Altogether, SECReTE motifs can be found in both TMD-containing and -lacking transcripts, whereby *NYN-*based motifs are contributed principally by the TMD regions of SRP-dependent secretome proteins. In contrast, *NNY-*based SECReTE motifs are enriched in soluble/SRP-independent secretome proteins. Finally, we note that SECReTE motifs are equally distributed to UTRs in the case of yeast and preferentially in the case humans, after normalization for gene length (S4A Fig and S6A Fig).

Although SECReTE10 enables the classification of mSMPs (Fig 1C and Fig 4C), the separation between secretome and non-secretome is not absolute and mRNAs coding for non-secretome proteins may also contain SECReTE sequences. While this might suggest that the motif is not completely defined, it might also imply that SECReTE plays a role in non-secretome mRNAs, perhaps in ER localization. There is an ongoing debate regarding whether mRNAs encoding cytosolic proteins localize to the ER and undergo translation by ER-associated ribosomes [46,47]. The idea that ER can support the translation of both secretory and cytosolic proteins was initially proposed by Nicchitta and colleagues [14–16,44,48]. Furthermore, they suggested that since translation initiation can start before the emergence of the signal sequence, ER-bound ribosomes would not distinguish between mRNAs and, therefore, mRNAs encoding cytosolic proteins can tether to ER membranes [16,23,44]. The fact that a large fraction of mRNAs encoding cytosolic proteins also contain SECReTE raises the possibility that their targeting to the ER is intentional and that this motif plays a role in it, even if the protein is not destined for secretion. Thus, SECReTE presence could be an organizing principle for transcript localization to the ER, while the presence or absence of an ER translocation signal (*e*.*g*. signal peptide, TMD, GPI anchor) in the polypeptide is the determinant for either secretion or cytosolic localization, respectively.

Gene ontology analysis revealed that genes encoding cell wall proteins are the most enriched with SECReTE (Fig 5A–5C). In contrast, TA-protein encoding transcripts show less (Fig 5C), perhaps since they are not enriched on ER membranes [22,23] and their translation products translocate to the ER only after full translation in the cytosol [32–34]. This finding implies that SECReTE is more abundant in mRNAs that are meant to be translated on (or near) the ER. Importantly, SECReTE was also identified with an unbiased method for motif discovery using the MEME server to find common sequence elements in cell wall genes. This parallel methodology supports our original identification of SECReTE and its importance is further enhanced by the discovery that it is present from bacteria to humans (Fig 4). As in yeast, human mSMPs are more enriched with SECReTE than non-secretome transcripts and this is independent of TMD presence (Fig 4B and 4C). Unlike yeast, however, human transcripts contain much larger UTR sequences and SECReTE elements appear to be more abundant therein, both in number and distribution (S6A Fig).

The physiological relevance of SECReTE was explored by altering its enrichment in three mSMPs: *SUC2*, *HSP150*, and *CCW12* (Fig 5 & Fig 6, and S7 Fig, S8 Fig, & S9 Fig). Although the amino acid sequences were not altered by motif mutation, the functionality of these genes was. *SUC2* SECReTE mutants exhibited altered growth rates on sucrose-containing medium in comparison to WT cells, *i*.*e*. reduced growth when motif score (number) was decreased and better growth when motif score was elevated (Fig 5A). Moreover, either the decrease or increase of motif score corresponded directly with a decrease or increase in invertase synthesis, invertase secretion, and the rate of secretion, respectively (Fig 5A–5C). *HSP150* SECReTE mutants also behaved differently, *i*.*e*. *HSP150*(-)SECReTE cells exhibited higher sensitivity to CFW in comparison to WT cells, while *HSP150*(+)SECReTE cells were more resistant (Fig 5D). Similarly, *CCW12*(-)SECReTE cells exhibited hypersensitivity to HB (Fig 5F). These findings strengthen the notion that SECReTE may play an important role in regulating the amount of protein secreted from cells. This idea was verified using an exogenous substrate, SSGAS-GFP, whose secretion was significantly enhanced upon addition of the *GAS1* 3’UTR containing the SECReTE motif (Fig 5G). The number of SECReTE motifs not only increased protein production and secretion, it also enhanced the localization of *SUC2* and *HSP150* transcripts to the ER (Fig 6 and S9B and S9C Fig). Thus, it would seem clear that SECReTE motifs enhance mRNA localization to the ER and subsequent secretome protein production and secretion, although the mechanism is not entirely known. It may be that SECReTE abundance helps stabilize secretome mRNAs as observed (S8 Fig and S9A Fig), perhaps by increasing their localization to the ER (Fig 6A–6C and S9B and S9C Fig), and through this mechanism yields higher amounts of protein translation and secretion. Higher levels of translation may promote the elevated rate of secretion afforded by (+)SECReTE mutations, although further work is necessary to fully resolve the function(s) of SECReTE.

Although SECReTE is present throughout evolution, it is not a strict sequence-based motif since a wide variety of pyrimidine-rich sequences fit its demands. This variability might allow for the preferential binding of specific mSMPs (or non-secretome-encoding mRNAs that contain SECReTE elements) to different SBPs under different conditions, depending upon secretory needs of the cells. While it is generally assumed that mRNA localization is required for local translation and proper positioning of the translated protein, SBP binding post-export may provide spatial and temporal regulation of mRNA stability and protein synthesis [49,50]. Moreover, additional features within secretome mRNAs may also influence both protein synthesis and secretion. For example, Palazzo *et al* (2007) previously showed that the low usage of adenine residues created no-A stretches within the signal sequence of SSCR-encoding proteins and the addition of adenines could affect nuclear export of the mRNA [29]. Thus, multiple *cis* RNA elements appear to impinge upon the translational control of secreted proteins.

Correct mRNA localization is not redundant to protein localization, but is yet another level of regulation that affects protein production. Supportive of this model is Puf3, an RBP that targets its associated mRNAs to the surface of the mitochondria [51]. In addition to its mitochondrial targeting role, Puf3 binding regulates the translational fate of mRNAs. Specifically, Puf3 binding leads to mRNA decay and repressed translation on high glucose, but becomes phosphorylated and promotes translation under low glucose conditions [52,53]. Interestingly, alterations in *SUC2* and *HSP150* SECReTE motifs also support this model, as mutations altered the amount of secreted protein, but not necessarily the ratio between secreted and non-secreted protein (Fig 5B and 5D). As Suc2 and Hsp150 both contain a signal peptide, SECReTE alteration does not necessarily affect protein targeting, but only mRNA targeting. Yet, if localizing mRNAs to the ER is important for conferring efficient translation, either through mRNA stabilization or the regulation of protein production, then SECReTE presence and strength (in terms of length or number) is expected to fill such a regulatory role. If SECReTE affects mRNA stabilization, this might well explain why we observed a decrease in *SUC2*(-)SECReTE mRNA levels and an increase in *SUC2*(+)SECReTE mRNA levels, in comparison to WT cells (Fig 6C and S8A Fig & S9A Fig). Moreover, we found that SECReTE abundance in *SUC2* and *HSP150* led to enhanced ER localization and membrane association (Figs 6 and S9B), suggesting that ER localization and mRNA stability are likely to be interconnected. Taken together, our results suggest that SECReTE abundance affects the localization, stability, and translation of secretome mRNAs (Fig 5, Fig 6, S8 Fig & S9 Fig).

If SECReTE is a *cis* regulatory element, the question is who are its *trans*-acting partners? Large-scale approaches have been used to identify mRNAs that interact with known RBPs in yeast [41–43]. These analyses enabled the identification of Bfr1, Whi3, Puf1, Puf2, Scp160, and Khd1 as potential SBPs, based upon their ability to interact with known SECReTE-containing transcripts (Fig 7A). As a means of verification, we first deleted individual RBPs and determined whether this alleviated the growth differences between WT and *HSP150*(+)SECReTE cells on CFW-containing medium, as might be expected upon the removal of a *bona fide* SBP. While the deletion of *PUF1*, *PUF2*, or *SHE2* did not alter the increased resistance of *HSP150*(+)SECReTE cells to CFW, those of *KHD1* and *WHI3* did (Fig 7D and S10 Fig). This suggests that they may be SBPs and several indications support the idea that Whi3 and Khd1 serve in this regard. For example, Whi3 possesses an RNA recognition motif and was already identified as preferentially binding mSMPs, including *HSP150* [41,54]. Whi3 also binds *CLN3* mRNA and is important for the efficient retention of Cln3 at the ER [55], as well as to destabilize *CLN3* and other mRNA targets [54]. In addition, the *whi3* deletion mutant is sensitive to cell wall perturbing agents, such as CFW and congo-Red [41], and is synthetic lethal with the deletion of *CCW12* in a synthetic genetic analysis screen [40]. Thus, Whi3 is an attractive candidate SBP. The same can be said for Khd1, which interacts with hundreds of transcripts including many mSMPs [42], and contains 3 K homology (KH) RNA-binding domains suggested to cooperatively recognize triplets of C/U-rich sequence elements [56]. These transcripts include *CCW12* [42] and, correspondingly, Khd1 plays a role in the cell wall integrity signaling pathway [57]. However, Khd1 is not essential and is best known for its association with *ASH1* mRNA and is required for both its translational repression and efficient localization to the bud tip [58]. *ASH1* mRNA, as well as mRNAs encoding polarity and secretion factors (*e*.*g*. *SRO7*), are physically bound to cortical ER and both are delivered to the bud tip via the same mechanism involving She2, She3, and Myo4/She1 [59,60]. Importantly, both *ASH1* and *SRO7* have SECReTE10 motifs (S3 Table). Thus, Khd1 interactions with SECReTE-containing mRNAs might potentiate their targeting to the ER, although this remains to be proven. Further work is required to identify SBPs and determine their role in protein secretion.

**Fig 8:**
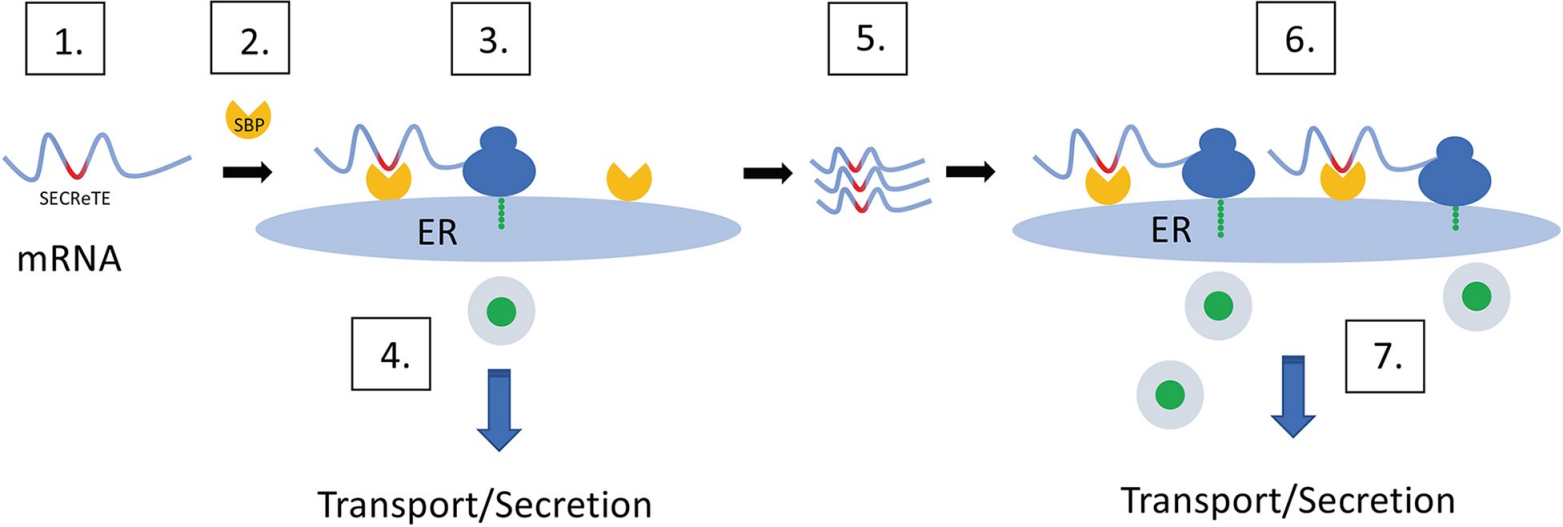
SECReTE plays an active role in protein secretion. SECReTE-containing transcripts (1) bind putative SECReTE-binding proteins (SBPs) (2) and induce mRNA targeting to the ER (3) and facilitate translation (connected small green dots = nascent chain polypeptide), translocation, and protein transport and secretion via the secretory pathway (4). Binding to an SBP or their association with the ER may confer mRNA stabilization (5), which in turn may further enhance association with the ER (6) and promote secretion (7) (large green dots = protein).

Although the mechanism is not entirely clear, SECReTE binding to ER-associated SBPs is likely to enhance transcript interactions with the ER and, thereby, increase mRNA stabilization, with the result being either increased translation efficiency and/or number of mRNAs translated on ER-bound ribosomes (see model, Fig 8). Our model supports the idea that mRNA plays an active role in its own targeting and this does not necessarily contradict the importance of co-translational localization, but rather provides another level of regulation. Thus, we believe that SECReTE plays an important physiological role in the fine-tuning of cellular secretion.

Being both a unicellular and eukaryotic organism, *S*. *cerevisiae* is advantageous for the production of recombinant proteins as it grows quickly, is easy to culture, and secretes post-translationally modified proteins into the extracellular medium, which can facilitate their purification. Moreover, *S*. *cerevisiae* is a generally recognized as a safe (GRAS) organism, which makes it favorable for use in the production of biopharmaceuticals [61,62]. Unfortunately, the natural capacity of *S*. *cerevisiae* secretory pathway is relatively limited and, thus, mechanisms that improve secreted protein production would be of significant benefit. Since SECReTE abundance increases protein production and secretion its use as an added RNA motif may prove to be a simple low-cost tool to improve recombinant protein production.

## Materials and methods

### Yeast strains and plasmids used

The yeast strains and plasmids used in this study are listed in S1 Table and S2 Table, respectively.

### Yeast strains, genomic manipulations, and growth conditions

Yeasts were grown at the indicated temperature either in a standard growth medium (1% Yeast Extract, 2% Peptone, 2% Dextrose) or synthetic medium containing 2% glucose [*e*.*g*., synthetic complete (SC) and selective SC dropout medium lacking an amino acid or nucleotide base] [63]. Deletion strains using the *NAT* antibiotic resistance gene in WT (BY4741) cells were created using standard LiOAc transformation procedures and with nourseothricin (100μg/ml) for selection on synthetic solid medium. For the creation of SECReTE mutant strains, SECReTE gene fragments were designed with the appropriate modifications, from the first to the last mutated base, and synthesized either as a gBlock (Integrated DNA Technologies, Inc., Coralville, IA, USA) or cloned into a pUC57-AMP vector (Bio Basic Inc.). Both (-)SECReTE and (+)SECReTE strains were generated. *SUC2*(-)SECReTE, *SUC2* (+)SECReTE and *CCW12*(-)SECReTE strains were constructed in the BY4741 background genome using the *delitto perfetto* method for genomic oligonucleotide recombination [64], in which the CORE cassette from pGKSU [64] was integrated first into the genomic region corresponding to site of the SECReTE gene fragment. The CORE cassette contains the *URA3* selection marker with an *I-SceI* homing endonuclease site and a separate inducible *I-SceI* gene. The SECReTE gene fragment for *CCW12*(-)SECReTE was amplified from the synthetic gBlock using primer sequences containing 20 bases of homology to both the region outside of the desired genomic locus and the CORE cassette. The amplified SECReTE gene fragment subsequently replaced the CORE cassette in the desired genomic site through an additional step of integration. CRISPR/Cas9 was utilized instead to generate the *HSP150* mutant strains. *HSP150*(-)SECReTE and *HSP150*(+)SECReTE were created in the BY4741 genome. The CRISPR/Cas9 procedure involved deletion of the native genomic region corresponding to the SECReTE gene fragment, using the *NAT* cassette from pFA6-NatMX6. A CRISPR/Cas9 plasmid vector was designed to express the Cas9 gene, a guide RNA that targets the *NAT* cassette, and the *LEU2* selection marker. The CRISPR/Cas9 plasmid was co-transformed with the amplified SECReTE gene fragment to replace the *NAT* cassette. Standard LiOAc-based protocols were employed for transformations of plasmids and PCR products into yeast. Transformed cells were then grown for 2–4 days on selective media. Correct integrations were verified at each step using PCR and, at the final step, accurate integration of the (-)SECReTE or (+)SECReTE sequences was confirmed by DNA sequencing.

### Quantitative RT-PCR (qRT-PCR)

RNA was extracted and purified from overnight cultures using a MasterPure Yeast RNA Purification kit (Epicentre Biotechnologies). For each sample, 2μg of purified RNA was treated with DNase (Promega, Madison, WI, USA) for 2hrs at 37°C and subjected to reverse transcription (RT) using Moloney murine leukemia virus RT RNase H(-) (Promega) under the recommended manufacturer conditions. Primer pairs were designed, using NCBI Primer-Blast [65], to produce only one amplicon (60-70bp). Standard curves were generated for each pair of primers and primer efficiency was measured. All sets of reactions were conducted in triplicate and each included a negative control (H_2_O). qRT-PCR was performed using a LightCycler480 device and SYBR Green PCR Master Mix (Applied Biosystems, Waltham, Massachusetts, USA). Two-step qRT-PCR thermocycling parameters were used as specified by the manufacturer. Analysis of the melting curve assessed the specificity of individual real-time PCR products and revealed a single peak for each real-time PCR product. The *ACT1* or *UBC6* RNAs were used for normalization and fold-change was calculated relative to WT cells.

### Drop test growth assays

Drop test assays were performed by growing yeast strains in YPD medium to mid-log phase and then performing serial dilution five times (10-fold each) in fresh medium. Cells were spotted onto plates with different conditions and incubated for 48hrs, prior to photo-documentation. Calcofluor White (CFW) or Hygromycin B (HB) sensitivity was tested by spotting cells onto YPD plates containing either 25μg/ml HB or 50μg/ml CFW (dissolved in DMSO, prepared as described) [66], following the protocol as mentioned above.

### Hsp150 and GFP secretion assays

For the induction of Hsp150 secretion, strains were grown in YPD overnight at 26°C, diluted in YPD medium to 0.2 O.D._600_ units, and then incubated at 37°C and grown until log-phase. For GFP secretion, yeast were grown O/N to 0.2 O.D._600_ at 30°C in synthetic selective medium containing raffinose as a carbon source, then diluted to 0.2 O.D._600_ units in YP-Gal and grown to mid-log phase (0.6–0.8 O.D._600_) at 30°C. Next, 1.8ml of the culture was taken from each strain and centrifuged for 3mins at 1900 x *g* at room temperature. Trichloroacetic Acid (100% w/v) protein precipitation was performed on the supernatant and protein extraction, using NaOH 0.1M, was performed on the pellet [67]. Samples were separated on SDS-PAGE gels, blotted electrophoretically onto nitrocellulose membranes, and detected by incubation with rabbit anti-Hsp150 [1:10,000 dilution; gift from Jussi Jäntti (VTT Research, Helsinki)] or monoclonal mouse anti-GFP (Roche Applied Science, Penzberg, Germany) antibodies followed by visualisation using the Enhanced Chemiluminescence (ECL) detection system with anti-rabbit peroxidase-conjugated antibodies (1:10,000, Amersham Biosciences). Protein markers (ExcelBand 3-color Broad Range Protein Marker PM2700, SMOBiO Technology, Inc., Hsinchu, Taiwan) were used to assess protein molecular mass.

### Invertase assay

Invertase secretion was measured as described previously [68]. Cell preparation for the invertase assay was performed as described in [69]. The protocol was optimized based on previous work [70]. Internal and external activities were expressed in units based on absorption at 540 nm (1 U = 1 μmol glucose released/min per OD unit).

### Single-molecule FISH

Yeast cells expressing Sec63-GFP were grown to mid-log phase and shifted to low glucose–containing medium [0.1% glucose] for 1.5 h to induce *SUC2* expression. Cells were fixed in the same medium upon the addition of paraformaldehyde (4% final concentration) and incubated at room temperature (RT) for 45min with rotation. Cells were gently washed three times with ice cold Buffer B (0.1M potassium phosphate buffer, pH 7.5 containing 1.2M sorbitol), after which cells were spheroplasted in 1ml of freshly prepared spheroplast buffer [Buffer B supplemented with 20mM ribonucleoside vanadyl complexes (Sigma-Aldrich, St. Louis, MO), 20mM β-mercaptoethanol, and lyticase (Sigma-Aldrich, St. Louis, MO) (25 U per O.D._600_ unit of cells)] for 10min at 30°C. The spheroplasts were centrifuged for 5min at 1300 × *g* at 4°C and washed twice in ice cold Buffer B. Spheroplasts were then resuspended in Buffer B and approximately 2.5 O.D._600_ units of cells were placed on poly-L-lysine coated coverslips in 12-well plates and incubated on ice for 30 min. Cells were carefully washed once with Buffer B, then incubated with 70% ethanol for several hours to overnight at -20°C. Afterwards, cells were washed once with SSCx2 (0.3M sodium chloride, 30mM sodium citrate), followed by incubating with Wash buffer (SSCx2 with 10% formamide), for 15 min at room temperature (RT; ~23°C). Next, 45μl of hybridization buffer (SSCx2, 10% dextran sulfate, 10% formamide, 2mM ribonucleoside vanadyl complexes, 1mg/ml E. coli tRNA, and 0.2mg/ml BSA) containing 250nM of the TAMRA-labeled Stellaris probe mix for native *SUC2* or *HSP150* (Biosearch Technologies, Novato, CA)) was placed on parafilm in a hybridization chamber. Coverslips with the immobilized cells were placed face down on top of the hybridization buffer and were incubated overnight at 37°C in the dark. After probe hybridization, cells were incubated twice in Wash buffer for 15min at 37°C. Cells were then washed once with SSCx2 containing 0.1% Triton X-100 and incubated with SSCx2 supplemented with 0.5μg/ml DAPI for 1min at RT and finally washed with SSCx2 for 5min at RT. Cells were mounted with Prolong Glass (Thermo Scientific) mounting media on clean microscope slides. Samples were imaged using a Zeiss AxioObserver Z1 DuoLink dual camera imaging system equipped with an Illuminator HXP 120 V light source, PlanApo 100× 1.4 NA oil immersion objective, and Hamamatsu Flash 4 sCMOS cameras. Incremental (0.2μm) *z*-stack images were taken using a motorized XYZ scanning stage 130x100 PIEZO and ZEN2 software at 0.0645μm/pixel. Images were processed by deconvolution. At least 50 cells each showing both cER and nER labeling, as well as mRNA spots, were scored for ER localization per cell type [*e*.*g*. native, (+)SECReTE, and (-)SECReTE] examined. Scoring of mRNA granules (spots) was performed using the FISH-quant program [71] (https://bitbucket.org/muellerflorian/fish_quant) to analyze deconvolved images of single cells. mRNA co-localization to cER or nER was scored manually and was defined by overlap between the deconvolved signals.

### Subcellular fractionation

Yeast cells were grown to mid-log phase (O.D._600_ = 0.6–0.8) and shifted to low glucose–containing medium (0.1% glucose) for 1.5hrs to induce *SUC2* expression. Cultures (400ml) were centrifuged for 3min at 500 × *g*, resuspended in a buffer solution containing 50mM Tris (pH 7.6), 150mM NaCl, 200U of RNasin Ribonuclease Inhibitor (Promega, Madison, WI), Complete Protease Inhibitor Cocktail (Roche Diagnostics, Basel, Switzerland), and cycloheximide 100μg/ml, and disrupted using glass beads and vortexing for 10min at 4°C. Crude lysates were centrifuged for 10min at 1000 × *g* to remove cell debris, and then 1ml of each lysate was subjected to ultracentrifugation for 1 h at 48,000 × *g*. The resulting pellet was then resuspended in 500μl of buffer containing 50mM Tris (pH 7.6), 150mM NaCl, 80U/ml RNasin Ribonuclease Inhibitor, Complete Protease Inhibitor Cocktail, and 100μg/ml cycloheximide. Next total RNA from both the membrane fraction (resuspended pellets) and the cytosolic fraction (supernatants) was isolated using the MasterPure Yeast RNA Purification Kit (including DNase I treatment) according to the manufacturer's recommendations.

### Computational analyses

#### Gene ontology

Definition of the secretome was used according to Ast *et al* [19]. This gene set includes all genes that contain at least one TMD and/or signal sequence and are not mitochondrial. The TMHMM tool [72] was used to define TMD-containing proteins. Cell wall and tail anchored genes were defined according to UniProt. Data from Jan *et al* [22] were used to define other groups of genes and for defining human GO terms. The GO Slim Mapper tool (SGD) (www.yeastgenome.org/cgi-bin/GO/goSlimMapper.pl) was used to classify SECReTE10- and SECReTE15-positive genes.

### Assignment of SECReTE and permutation analysis

To calculate the SECReTE count for each gene, the length of an uninterrupted run of *NNY* triplet repeats was counted separately for each of the three different codon positions (*i*.*e*. *YNN*, *NYN*, *NNY*, where *N* is any nucleotide and *Y* is a pyrimidine) for every gene. A SECReTE motif defined by the number of *NNY* triplet repeats where the threshold is ≥10 (see Results). Note that a gene may have more than one SECReTE motif along its length, each potentially in a different frame (relative to the coding sequence). The SECReTE count is defined as the number of SECReTE motifs present in a transcript.

For permutation analysis, the codon order of each gene sequence was randomly shuffled 1,000 times to evaluate the statistical significance of the SECReTE count in random sequences with unchanged codon usage. A *Z* score was calculated for each motif according to the formula: *Z* = (Observed–mean)/STD. Observed is the length of the motif in the real gene sequence. Mean is the average SECReTE length for all shuffled sequences of the gene. STD is the standard deviation of the SECReTE length from all shuffled sequences of the gene.

### Calculation of RRY motif scores

In order to differentiate between SECReTE motifs that follow an *NNY* pattern and those that are simply pyrimidine-rich, an *RRY [R* (purine); G or A)] score was calculated for each defined motif. Since poly-Y conforms to *NNY*, *RRY* was used instead. The score is calculated for every motif by representing each nucleotide of the motif that follows the *RRY* pattern as ‘1’ and each nucleotide that defies this pattern as ‘0’, and taking the average score over the entire length of the motif. The score is calculated for all three possible frames and the maximal score between them is taken for each motif. The UTR sequences for the yeast genome were obtained from: https://downloads.yeastgenome.org/sequence/S288C_reference/.

### Identification of a motif in cell wall proteins

A motif search was performed by MEME suites [73] (http://meme-suite.org/tools/meme) to identify RNA motifs in genes encoding cell wall proteins in yeast.

## Supporting information

S1 Table Yeast strains used in this study. (DOCX)

S2 Table Plasmids used in this study. (DOCX)

S3 Table Genome-wide scoring of SECReTE motifs and positions in yeast genes. *SECReTE10 scores–CDS* spread sheet: Yeast (*S*. *cerevisiae*) genes were scored according to the number of SECReTE10 and above (*i*.*e*. ≥10) motifs and the reading frame in the coding region (CDS) in which they appear (*i*.*e*. 1^st^—*YNN*, 2^nd^—*NYN*, 3^rd^—*NNY*). Information regarding CDS length, whether the gene is encodes a secretome protein or not, and if the gene contains a transmembrane region (TMD) is listed. *SECReTE10-CDS* spread sheet: The start location for each SECReTE motif, number of triplet nucleotide repetitions, nucleotide sequence of that motif, and whether the motif overlaps with a TMD is provided for each yeast CDS. *SECReTE10-5’UTR* spread sheet: The start location for each SECReTE motif in the 5’UTR, number of triplet nucleotide repetitions, nucleotide sequence of that motif, and whether the gene encodes a secretome protein with or without a TMD is listed. *SECReTE10-3’UTR* spread sheet: The start location for each SECReTE motif in the 3’UTR, number of triplet nucleotide repetitions, nucleotide sequence of that motif, and whether the gene encodes a secretome protein with or without a TMD is listed.
(XLSX)

S4 Table Genome-wide scoring of SECReTE motifs and position in human genes. SECReTE10 and above motifs were identified for each human (*H*. *sapiens*) gene and are listed in the spread sheets by position in the coding region (*CDS*) or untranslated regions (UTRs; *3’UTR* and *5’UTR*). The consensus CDS (CCDS) name for each gene is listed, along with the Uniprot and general gene nomenclature names. *CDS—*SECReTE length (# triplet nucleotide repetitions), position (frame), location in CDS, motif sequence, and whether the gene is a secretome protein bearing a transmembrane domain (TMD)-containing region is listed. *5’UTR*—SECReTE length (# triplet nucleotide repetitions), location in 5’UTR, motif sequence, and whether the gene encodes a secretome protein bearing a transmembrane domain (TMD is listed. *3’UTR*—SECReTE length (# triplet nucleotide repetitions), location in 3’UTR, motif sequence, and whether the gene encodes a secretome protein bearing a transmembrane domain (TMD) is listed.
(XLSX)

S5 Table SECReTE score alteration in SUC2, HSP150, and CCW12. (DOCX)

S1 Fig SECReTE-containing transcripts are more enriched in ER, but not mitochondrial, fractions. Cumulative distribution of mRNA enrichment was plotted separately for SECReTE-containing transcripts and transcripts lacking SECReTE. **(A) Transcripts containing SECReTE are more abundant on ER-bound ribosomes.** A plot of the enrichment data obtained from proximity-specific ribosome profiling with BirA-Ubc6 (2min; cycloheximide–CHX) [22]. **(B) Transcripts with SECReTE are more abundant on membranal polysomes.** A plot of the enrichment data obtained from the ribosome profiling of polysomes extracted from the membrane fraction of yeast cells [23]. **(C) Transcripts with SECReTE are more abundant in the membrane fraction.** A plot of the enrichment data obtained from RNA-seq analysis of the membrane fraction of yeast cells [23]. **(D) Transcripts with SECReTE are not abundant on mitochondrial ribosomes.** A plot of the enrichment data obtained from proximity-specific ribosome profiling with BirA-Om45 (2min; CHX) [22]. **(E) Removal of the TMD regions does not alter the enrichment of SECReTE motifs in mRNAs coding for TMD-containing secretome proteins than for non-secretome TMD-containing proteins.** SECReTE presence, according to the indicated threshold, was counted in mRNAs coding for TMD-encoding secretome (blue) and non-secreted (orange) proteins in which the TMD regions were first removed. Bars represent the fraction of SECReTE positive transcripts at the indicated threshold. SECReTE abundance remains significantly higher in the TMD-encoding secretome mRNAs. *p < 0.008 (secretome vs. non-secretome; SECReTE7); <0.015 (SECReTE10); <0.05 (SECReTE12), chi-square.
(TIF)

S2 Fig Transcripts coding for SRP-independent proteins are enriched with SECReTE. **(A-C) SECReTE10 distribution in groups of genes separated according to their SRP-dependence predictions.** Genes were separated according to prediction of their SRP-dependence [19] in the dataset of Costa et al [24]. Box-plots represent the distribution of SECReTE10 in each of the coding positions in the different groups: *Int—*internal TMD; *NS–*non-secretome; *SRP dep–*SRP-dependent; *SRP indep–*SRP-independent; *TA—*tail- anchored. **(A) SECReTE10 distribution at the first codon position.** As was shown, SECReTE10 is barely present in the first codon position of all genes. **(B) SECReTE10 distribution at the second codon position.** Secretome groups are enriched with SECReTE10 at the second codon position. Among secretome genes those with an internal TMD (*Int*) are the most enriched, while tail-anchored genes are the least enriched. **(C) SECReTE10 distribution at the third codon position.** SECReTE10 at the third position is more enriched in SRP-independent genes (*SRP indep*) than in SRP-dependent genes (*SRP dep*). Genes with an internal TMD (*Int*) are also enriched with SECReTE10 at the third position of the codon. **(D) SECReTE is enriched in mRNAs that are ER-bound after SRP-depletion.** Secretome transcripts were separated according to SECReTE10 presence or absence at the third position of the codon upon auxin-induced SRP depletion. SECReTE10-containing genes = light blue; SECReTE10-lacking genes = pink. Density plots were obtained from data of a time course of SRP depletion using proximity-specific ribosome profiling with Sec63-BirA in the presence of CHX. **(E-F) mRNAs encoding cell wall proteins remain ER-bound after SRP-depletion.** Cell component ontology analysis revealed that transcripts remaining on the ER after 60min of SRP depletion [log2(Sec63-BirA)>2] are highly enriched with cell wall proteins (enrichment is 2.8-fold higher than mSMPs (E) and 14.3-fold higher than all yeast genes (F).
(TIF)

S3 Fig Repetitive NNR and NNW motifs are present in non-secretome transcripts lacking transmembrane domains. Computational analysis of ≥10 repetitive *NNX* motifs in the coding region (CDS) of secretome and non-secretome transcripts, either with (A) or without (B) the transmembrane domains (TMD), respectively, is shown. *X* = K (T/G), M (C/A), R (A/G), S (G/C), or W (A/T)]. *NNR* and *NNW* motifs are significantly more abundant in non-secretome genes without transmembrane domains (*p* = 1.3e-9 and 1.8e-5, chi-square after false discovery rate correction, respectively).
(TIF)

S4 Fig **SECReTE distribution and content (A) SECReTE is evenly distributed over the coding and UTR regions in yeast.** SECReTE distribution was plotted over the different gene regions (*e*.*g*. CDS, 5’UTR, and 3’UTR) of the yeast (left). Most SECReTE motifs (*i*.*e*. ≥10 *NNY*) are found in the CDS (left). However, normalization of SECReTE abundance over the mean region length shows that SECReTE is more or less evenly distributed between regions. **(B) SECReTE is uniformly distributed in genes expressing TMD-containing secretome proteins, but is somewhat more prevalent at the 5’ end of genes encoding soluble secretome proteins**. SECReTE motif (*i*.*e*. ≥10) distribution along the length of genes encoding secretome proteins was scored for both soluble and TMD-containing proteins before and after removal of the signal sequence-encoding region (SSCR:—*SP*). *Count =* number of genes with motif in delimited region. *Location* = location of motif along normalized gene length. Top four graphs illustrate SECReTE distribution in the full length genes including the UTR regions (Full). Bottom six graphs show the SECReTE count scored according to frame (*i*.*e*. *NNY* and *NYN*) in the coding regions, either before or after removal of the SSCR (- SP). The results show that SECReTE is uniformly distributed in TMD-containing proteins, but that the SSCR can contribute the motif in a subset of ~60 genes encoding soluble secreted proteins. **(C) SECReTE motifs in the CDS follow an *RRY* pattern, while UTR motifs are pyrimidine-rich.** The *RRY* score (see Methods) of SECReTE (≥10 *NNY*) residing in the CDS and UTRs of yeast genes were determined. *RRY-*based SECReTE motifs are significantly higher than those residing in the UTR (left) (unpaired t-test, *p* value <10^−25^). The pyrimidine content of SECReTE in the gene regions was also scored (right) and the UTR-based motifs show a higher Y content (unpaired t-test,p-value < 10^−25^). **(D) UTRs of secretome-encoding genes are enriched with pyrimidine compared to non-secretome-encoding genes.** The level of Y content in the 5’UTRs (left) and 3’UTRs (right) of secretome genes (blue) and non-secretome genes (grey) is plotted. The Y-content is significantly higher for both UTRs in secretome genes (unpaired t-test: 5’UTR *p* value = 4 x 10^−4^; 3’UTR *p* value = 5 x 10^−4^). **(E) Pyrimidine enrichment in the UTRs of secretome-encoding genes is due to SECReTE motifs.** Genes bearing SECReTE in their UTRs were removed prior to calculation of Y content of the UTRs. The result shows that there is no significant pyrimidine enrichment in the UTRs of secretome genes *per se* once SECReTE is removed (5’UTR *p* value = 0.9, 3’UTR *p* value = 0.9).
(TIF)

S5 Fig SECReTE abundance is not dependent on codon usage. Permutation analysis was conducted to evaluate the dependency of SECReTE on codon usage. To do that, codon composition was kept and sequences were randomly reshuffled 1000 times. The Z-score was calculated for each gene to assess the probability of the SECReTE10 to appear randomly (for Z-score calculation, see Materials and Methods). The higher the Z-score the less likely it is for SECReTE to appear randomly. **(A) SECReTE enrichment in secretome-encoding mRNAs is independent of codon usage.** Distribution plots of Z-scores show higher values for mRNAs encoding secretome proteins than for non-secretome proteins. **(B) SECReTE enrichment in mRNAs encoding both soluble and membranal secretome transcripts is independent of codon usage**. Distribution plots of Z-scores show higher values for mRNAs encoding secretome proteins (mSMPs; either with or without a TMD) than for non-secretome proteins (*i*.*e*. with or without a TMD). **(C) SECReTE enrichment in the second and third position of the codon is independent of codon usage.** The fraction of significant Z-scores (*i.e.* ≥1.96) is larger for mRNAs encoding secretome proteins than for non-secretome proteins. **(D) SECReTE enrichment in the second and third position of the codon is independent of both codon usage and TMD presence.** The fraction of significant Z-scores (*i.e.* ≥1.96) is larger for mRNAs encoding secretome proteins than for non-secretome proteins, either with or without a TMD.
(TIF)

S6 Fig SECReTE is mainly distributed to the UTR regions in humans. **A) Human SECReTE motifs are mainly UTR-localized.** Computational analysis of SECReTE (≥10 *NNY*) distribution over the different gene regions [5’UTR, 3’UTR, and coding region (CDS)] of humans, either without (left) or with (right) normalization for region length (based upon mean length of the region) is shown. (**B) Human SECReTE motifs in the CDS follow the *RRY* pattern, while the UTRs are pyrimidine-rich.** Computational analysis of *RRY* (≥10 *RRY*) enrichment (left) or pyrimidine content (right) for the different gene regions is shown. *RRY* repeat scoring (see Methods) of SECReTE motifs residing in the CDS is significantly higher than that of UTR-based SECReTE motifs (left; unpaired t-test, *p* value < 10^−100^). Yet, UTR-based motifs have a significantly higher Y-content (right; unpaired t-test, *p* value < 10^−100^).
(TIF)

S7 Fig Illustration of SECReTE and introduced SECReTE mutations in SUC2, HSP150, and CCW12. Graphs compare the number of triplet *NNY* repeats found along the length of the coding region of each gene either with (lower schematics) or without using a threshold of 10 consecutive *NNY* repeats (upper schematics) in the native and mutant SECReTE genes. **(A)** *SUC2*. **(B)** *HSP150*. **(C)** *CCW12*.
(TIF)

S8 Fig Mutations in SECReTE do not necessarily affect mRNA levels. mRNA levels of native or mutant *SUC2*, *CCW12*, and *HSP150* in the indicated strains were quantified by qRT-PCR. Fold-change was calculated relative to WT levels. **(A) *SUC2* mRNA levels are altered by SECReTE mutation**. Cells were grown to mid-log phase on SC medium containing 2% glucose at 30°C prior to shifting cells to low glucose medium for 1.5hrs. After harvesting and RNA extraction, primers used for amplifying the long transcript of *SUC2*, which encodes the secreted protein. Primers for actin were used for normalization. *SUC2*(-)SECReTE cells exhibited lower *SUC2* mRNA levels than WT, while *SUC2*(+)SECReTE cells yielded higher levels. Error bars represent the standard deviation of three biological repeats. **(B) *HSP150* mRNA levels are not altered by SECReTE mutation.** Yeast strains were grown to mid-log phase at either 26°C (red) or 37°C (blue) on YPD medium prior to harvesting and RNA extraction. *UBC6* was used for normalization. *HSP150* mRNA levels were not significantly changed as a result of SECReTE alterations. (**C) *CCW12* mRNA levels are not altered by SECReTE mutation.** Cells were grown to mid-log phase onYPD medium at 30°C prior to harvesting and RNA extraction. Primers used for amplifying *UBC6* were used for normalization. *CCW12* mRNA levels were not significantly changed as a result of SECReTE alterations.
(TIF)

S9 Fig smFISH labeling indicates that SECReTE abundance can influence mRNA granule count and localization of HSP150 mRNA to the ER. **(A) mRNA granule scores per cell measured using FISH-quant.** The number of mRNA granules (spots) detected by smFISH using Stellaris probes for *SUC2* and *HSP150* was scored for WT, (+)SECReTE (S+) and (-)SECReTE (S-) cells using FISH-quant (https://bitbucket.org/muellerflorian/fish_quant). The numbers correspond to the average number of mRNAs detected per cell ±SEM for the data shown in Fig 6A and 6B, and S9B and S9C Fig. **(B) Visualization of endogenously expressed *SUC2*(+)SECReTE and *SUC2*(-)SECReTE mRNAs using smFISH**. Yeast endogenously expressing WT *SUC2*, *SUC2*(+)SECReTE, or *SUC2*(-)SECReTE and Sec63-GFP from a plasmid were grown to mid-log phase on SC medium containing 2% glucose prior to shifting cells to low glucose-containing medium (0.05% glucose) to induce *SUC2* expression. Cells were processed for smFISH labeling using non-overlapping, TAMRA-labeled, FISH probes complementary to *SUC2*, prior to labeling with DAPI, as shown in Fig 6A. Additional representative images are shown in this figure. Line = 1μm. **(C) Visualization of endogenously expressed *HSP150*(+)SECReTE and *HSP150*(-)SECReTE mRNAs using smFISH**. Yeast endogenously expressing WT *HSP150*, *HSP150*(+)SECReTE, or *HSP150*(-)SECReTE and Sec63-GFP from a plasmid were grown to mid-log phase on SC medium prior to fixation. Cells were processed for smFISH labeling using non-overlapping, TAMRA-labeled, FISH probes complementary to *HSP150*, and stained with DAPI (shown in *merge*). mRNA granule scoring was performed using the FISH-quant algorithm and co-localization of the granules to the ER (both cER and nER) is shown in Fig 6B.
(TIF)

S10 Fig Identification of potential SECReTE-binding proteins. WT cells and either WT or *HSP150*(+)SECReTE cells deleted for genes encoding the indicated RBPs [*e*.*g*. Puf2, She2 (A) and Puf1(B)] were grown to mid-log phase on YPD at 30°C, prior to serial dilution and plating onto either solid YPD medium or YPD containing CFW. Yeast were grown 2 days prior to photo-documentation.
(TIF)

## References

[1] Martin KC, Ephrussi A. mRNA Localization: Gene Expression in the Spatial Dimension. Cell. 2009;136: 719–730. 10.1016/j.cell.2009.01.044 19239891 PMC2819924

[2] Buxbaum AR, Haimovich G, Singer RH. In the right place at the right time: visualizing and understanding mRNA localization. Nat Rev Mol Cell Biol. 2015;16: 95–109. 10.1038/nrm3918 25549890 PMC4484810

[3] Blobel G, Dobberstein B. Transfer of proteins across membranes. I. Presence of proteolytically processed and unprocessed nascent immunoglobulin light chains on membrane bound ribosomes of murine myeloma. J Cell Biol. 1975;67: 835–851. 10.1083/jcb.67.3.835 811671 PMC2111658

[4] Gilmore R, Blobel G, Walter P. Protein translocation across the endoplasmic reticulum.I. Detection in the Microsomal Membrane of a Receptor for the Signal Recognition Particle. J Cell Biol. 1982;95: 463–469. 10.1083/jcb.95.2.463 6292235 PMC2112970

[5] Walter P, Blobel G. Translocation of proteins across membranes III. Signal recognition protein (SRP) causes signal sequence- dependent and site specific arrest of chain elongation that is released by microsomal membranes. J Cell Biol. 1981;91: 557–561. 10.1083/jcb.91.2.557 7309797 PMC2111983

[6] Schwartz TU. Origins and evolution of cotranslational transport to the ER. Adv Exp Med Biol. 2007;607: 52–60. 10.1007/978-0-387-74021-8_4 17977458

[7] Saraogi I, Shan S. Molecular mechanism of co-translational protein targeting by the signal recognition particle. Traffic. 2011;12: 535–542. 10.1111/j.1600-0854.2011.01171.x 21291501 PMC3077218

[8] Kraut-Cohen J, Gerst JE. Addressing mRNAs to the ER: cis sequences act up! Trends Biochem Sci. 2010;35: 459–469. 10.1016/j.tibs.2010.02.006 20346679

[9] Weis BL, Schleiff E, Zerges W. Protein targeting to subcellular organelles via mRNA localization. Biochim Biophys Acta—Mol Cell Res. 2013;1833: 260–273. 10.1016/j.bbamcr.2012.04.004 23457718

[10] Mutka SC, Walter P. Multifaceted Physiological Response Allows Yeast to Adapt to the Loss of the Signal Recognition Particle-dependent Protein-targeting Pathway. Mol Biol Cell. 2001;12: 577–588. 10.1091/mbc.12.3.577 11251072 PMC30965

[11] Ren Y-G, Wagner KW, Knee DA, Aza-Blanc P, Nasoff M, Deveraux QL. Differential regulation of the TRAIL death receptors DR4 and DR5 by the signal recognition particle. Mol Biol Cell. 2004;15: 5064–5074. 10.1091/mbc.E04-03-0184 15356269 PMC524775

[12] Diehn M, Eisen MB, Botstein D, Brown PO. Large-scale identification of secreted and membrane-associated gene products using DNA microarrays. Nat Genet. 2000;25: 58–62. 10.1038/75603 10802657

[13] Lerner RS, Seiser RM, Zheng T, Lager PJ, Reedy MC, Keene JD, et al Partitioning and translation of mRNAs encoding soluble proteins on membrane-bound ribosomes. RNA. 2003;9: 1123–1137. 10.1261/rna.5610403 12923260 PMC1370476

[14] Pyhtila B, Zheng T, Lager PJ, Keene JD, Reedy MC, Nicchitta C V. Signal sequence- and translation-independent mRNA localization to the endoplasmic reticulum. RNA. 2008;14: 445–453. 10.1261/rna.721108 18192611 PMC2248262

[15] Reid DW, Nicchitta C V. Primary role for endoplasmic reticulum-bound ribosomes in cellular translation identified by ribosome profiling. J Biol Chem. 2012;287: 5518–5527. 10.1074/jbc.M111.312280 22199352 PMC3285328

[16] Jagannathan S, Reid DW, Cox AH, Jagannathan S, Reid DW, Cox AH, et al De novo translation initiation on membrane-bound ribosomes as a mechanism for localization of cytosolic protein mRNAs to the endoplasmic reticulum. RNA. 2014;20: 1489–1498. 10.1261/rna.045526.114 25142066 PMC4174431

[17] Chen Q, Jagannathan S, Reid DW, Zheng T, Nicchitta C V. Hierarchical regulation of mRNA partitioning between the cytoplasm and the endoplasmic reticulum of mammalian cells. Mol Biol Cell. 2011;22: 2646–2658. 10.1091/mbc.E11-03-0239 21613539 PMC3135488

[18] Kraut-Cohen J, Afanasieva E, Haim-Vilmovsky L, Slobodin B, Yosef I, Bibi E, et al Translation- and SRP-independent mRNA targeting to the endoplasmic reticulum in the yeast Saccharomyces cerevisiae. Mol Biol Cell. 2013;24: 3069–84. 10.1091/mbc.E13-01-0038 23904265 PMC3784381

[19] Ast T, Cohen G, Schuldiner M. A network of cytosolic factors targets SRP-independent proteins to the endoplasmic reticulum. Cell. 2013;152: 1134–1145. 10.1016/j.cell.2013.02.003 23452858

[20] Aviram N, Ast T, Costa EA, Arakel EC, Chuartzman SG, Jan CH, et al The SND proteins constitute an alternative targeting route to the endoplasmic reticulum. Nature. 2016;540: 134–138. 10.1038/nature20169 27905431 PMC5513701

[21] Johnson N, Powis K, High S. Post-translational translocation into the endoplasmic reticulum. Biochim Biophys Acta—Mol Cell Res. 2013;1833: 2403–2409. 10.1016/j.bbamcr.2012.12.008 23266354

[22] Jan CH, Williams CC, Weissman JS. Principles of ER cotranslational translocation revealed by proximity-specific ribosome profiling. Science. 2014;346: 748–751. 10.1126/science.1257522 25378630 PMC4285348

[23] Chartron JW, Hunt KCL, Frydman J. Cotranslational signal-independent SRP preloading during membrane targeting. Nature. 2016;536: 224–228. 10.1038/nature19309 27487213 PMC5120976

[24] Costa EA, Subramanian K, Nunnari J, Weissman JS. Defining the physiological role of SRP in protein-targeting efficiency and specificity. Science. 2018;359: 689–692. 10.1126/science.aar3607 29348368 PMC5970945

[25] Shahbabian K, Chartrand P. Control of cytoplasmic mRNA localization. Cell Mol Life Sci. 2012;69: 535–552. 10.1007/s00018-011-0814-3 21984598 PMC11115051

[26] Hamilton RS, Davis I. Identifying and searching for conserved RNA localisation signals. Methods Mol Biol. 2011;714: 447–466. 10.1007/978-1-61779-005-8_27 21431757 PMC3082378

[27] Polyansky A a, Hlevnjak M, Zagrovic B. Analogue encoding of physicochemical properties of proteins in their cognate messenger RNAs. Nat Commun. 2013;4: 2784 10.1038/ncomms3784 24253588 PMC3868254

[28] Cui X a., Palazzo AF. Localization of mRNAs to the endoplasmic reticulum. Wiley Interdiscip Rev RNA. 2014;5: 481–492. 10.1002/wrna.1225 24644132

[29] Palazzo AF, Springer M, Shibata Y, Lee C-S, Dias AP, Rapoport TA. The signal sequence coding region promotes nuclear export of mRNA. PLoS Biol. 2007;5: 2862–2874. 10.1371/journal.pbio.0050322 18052610 PMC2100149

[30] Wolfenden R V, Cullis PM, Southgate CC. Water, protein folding, and the genetic code. Science. 1979;206: 575–577. 10.1126/science.493962 493962

[31] Prilusky J, Bibi E. Studying membrane proteins through the eyes of the genetic code revealed a strong uracil bias in their coding mRNAs. Proc Natl Acad Sci U S A. 2009;106: 6662–6666. 10.1073/pnas.0902029106 19366666 PMC2672546

[32] Sharp PM, Li WH. The codon Adaptation Index—a measure of directional synonymous codon usage bias, and its potential applications. Nucleic Acids Res. 1987;15: 1281–95. 10.1093/nar/15.3.1281 3547335 PMC340524

[33] Denic V. A portrait of the GET pathway as a surprisingly complicated young man. Trends Biochem Sci. 2012;37: 411–417. 10.1016/j.tibs.2012.07.004 22951232 PMC3459580

[34] Stefanovic S, Hegde RS. Identification of a Targeting Factor for Posttranslational Membrane Protein Insertion into the ER. Cell. 2007;128: 1147–1159. 10.1016/j.cell.2007.01.036 17382883

[35] Cui XA, Zhang H, Palazzo AF. p180 promotes the ribosome-independent localization of a subset of mRNA to the endoplasmic reticulum. PLoS Biol. Public Library of Science 2012;10: e1001336 10.1371/journal.pbio.1001336 22679391 PMC3362647

[36] Hsu P-H, Chiang P-C, Liu C-H, Chang Y-W. Characterization of Cell Wall Proteins in Saccharomyces cerevisiae Clinical Isolates Elucidates Hsp150p in Virulence. PLoS One. 2015;10: e0135174 10.1371/journal.pone.0135174 26270963 PMC4535956

[37] Russo P, Simonen M, Uimari A, Teesalu T, Makarow M. Dual regulation by heat and nutrient stress of the yeast HSP150 gene encoding a secretory glycoprotein. Mol Gen Genet. 1993;239: 273–80. 8510655 10.1007/BF00281628

[38] Russo P, Kalkkinen N, Sareneva H, Paakkola J, Makarow M. A heat shock gene from Saccharomyces cerevisiae encoding a secretory glycoprotein. Proc Natl Acad Sci U S A. 1992;89: 3671–5. 10.1073/pnas.89.9.3671 1570286 PMC525552

[39] Ragni E, Sipiczki M, Strahl S. Characterization of Ccw12p, a major key player in cell wall stability of Saccharomyces cerevisiae. Yeast. 2007;24: 309–319. 10.1002/yea.1465 17315267

[40] Ragni E, Piberger H, Neupert C, García-Cantalejo J, Popolo L, Arroyo J, et al The genetic interaction network of CCW12, a Saccharomyces cerevisiae gene required for cell wall integrity during budding and formation of mating projections. BMC Genomics. 2011;12: 107 10.1186/1471-2164-12-107 21320323 PMC3049148

[41] Colomina N, Ferrezuelo F, Wang H, Aldea M, Garí E. Whi3, a developmental regulator of budding yeast, binds a large set of mRNAs functionally related to the endoplasmic reticulum. J Biol Chem. 2008;283: 28670–28679. 10.1074/jbc.M804604200 18667435 PMC2661415

[42] Hasegawa Y, Irie K, Gerber AP. Distinct roles for Khd1p in the localization and expression of bud-localized mRNAs in yeast. RNA. 2008;14: 2333–2347. 10.1261/rna.1016508 18805955 PMC2578860

[43] Hogan DJ, Riordan DP, Gerber AP, Herschlag D, Brown PO. Diverse RNA-binding proteins interact with functionally related sets of RNAs, suggesting an extensive regulatory system. PLoS Biol. 2008;6: 2297–2313. 10.1371/journal.pbio.0060255 18959479 PMC2573929

[44] Reid DW, Nicchitta C V. Diversity and selectivity in mRNA translation on the endoplasmic reticulum. Nat Rev Neurosci. 2015;16: 221–231. 10.1038/nrm3958 25735911 PMC4494666

[45] Cui XA, Zhang H, Palazzo AF, Fugate RD, Reichlin M. p180 Promotes the Ribosome-Independent Localization of a Subset of mRNA to the Endoplasmic Reticulum. PLoS Biol. 2012;10: e1001336 10.1371/journal.pbio.1001336 22679391 PMC3362647

[46] Reid DW, Nicchitta C V. Comment on “Principles of ER cotranslational translocation revealed by proximity-specific ribosome profiling.” Science (80-). 2015;348. 10.1126/science.aaa7257 PMC4862200 26068841

[47] Jan CH, Williams CC, Weissman JS. Response to Comment on “Principles of ER cotranslational translocation revealed by proximity-specific ribosome profiling.” Science (80-). 2015;348. 10.1126/science.aaa8299 26068842

[48] Gerst JE. Message on the web: mRNA and ER co-trafficking. Trends Cell Biol. 2008;18: 68–76. 10.1016/j.tcb.2007.11.005 18215524

[49] Kejiou NS, Palazzo AF. mRNA localization as a rheostat to regulate subcellular gene expression. Wiley Interdiscip Rev RNA. 2017;8: e1416 10.1002/wrna.1416 28120379

[50] Chin A, Lécuyer E. RNA localization: Making its way to the center stage. Biochimica et Biophysica Acta—General Subjects. 10.1016/j.bbagen.2017.06.011 28630007

[51] Saint-Georges Y, Garcia M, Delaveau T, Jourdren L, Le Crom S, Lemoine S, et al Yeast Mitochondrial Biogenesis: A Role for the PUF RNA-Binding Protein Puf3p in mRNA Localization. PLoS One. 2008;3: e2293 10.1371/journal.pone.0002293 18523582 PMC2387061

[52] Houshmandi SS, Olivas WM. Yeast Puf3 mutants reveal the complexity of Puf-RNA binding and identify a loop required for regulation of mRNA decay. RNA. 2005;11: 1655–66. 10.1261/rna.2168505 16244132 PMC1370852

[53] Olivas W, Parker R. The Puf3 protein is a transcript-specific regulator of mRNA degradation in yeast. EMBO J. 2000;19: 6602–11. 10.1093/emboj/19.23.6602 11101532 PMC305854

[54] Cai Y, Futcher B, Waern K, Shou C, Raha D. Effects of the Yeast RNA-Binding Protein Whi3 on the Half-Life and Abundance of CLN3 mRNA and Other Targets. Gladfelter AS, editor. PLoS One. 2013;8: e84630 10.1371/journal.pone.0084630 24386402 PMC3875557

[55] Vergés E, Colomina N, Garí E, Gallego C, Aldea M. Cyclin Cln3 Is Retained at the ER and Released by the J Chaperone Ydj1 in Late G1 to Trigger Cell Cycle Entry. Mol Cell. 2007;26: 649–662. 10.1016/j.molcel.2007.04.023 17560371

[56] Paziewska A, Wyrwicz LS, Bujnicki JM, Bomsztyk K, Ostrowski J. Cooperative binding of the hnRNP K three KH domains to mRNA targets. FEBS Lett. 2004;577: 134–140. 10.1016/j.febslet.2004.08.086 15527774

[57] Ito W, Li X, Irie K, Mizuno T, Irie K. RNA-Binding Protein Khd1 and Ccr4 Deadenylase Play Overlapping Roles in the Cell Wall Integrity Pathway in Saccharomyces cerevisiae. Eukaryot Cell. 2011;10: 1340–1347. 10.1128/EC.05181-11 21873511 PMC3187069

[58] Irie K, Tadauchi T, Takizawa PA, Vale RD, Matsumoto K, Herskowitz I. The Khd1 protein, which has three KH RNA-binding motifs, is required for proper localization of ASH1 mRNA in yeast. EMBO J. 2002;21: 1158–67. 10.1093/emboj/21.5.1158 11867544 PMC125877

[59] Aronov S, Gelin-Licht R, Zipor G, Haim L, Safran E, Gerst JE. mRNAs Encoding Polarity and Exocytosis Factors Are Cotransported with the Cortical Endoplasmic Reticulum to the Incipient Bud in Saccharomyces cerevisiae. Mol Cell Biol. 2007;27: 3441–3455. 10.1128/MCB.01643-06 17339339 PMC1899969

[60] Schmid M, Jaedicke A, Du T-G, Jansen R-P. Coordination of Endoplasmic Reticulum and mRNA Localization to the Yeast Bud. Curr Biol. 2006;16: 1538–1543. 10.1016/j.cub.2006.06.025 16890529

[61] Tang H, Song M, He Y, Wang J, Wang S, Shen Y, et al Engineering vesicle trafficking improves the extracellular activity and surface display efficiency of cellulases in Saccharomyces cerevisiae. Biotechnol Biofuels. 2017;10: 53 10.1186/s13068-017-0738-8 28261326 PMC5327580

[62] Roohvand F, Shokri M, Abdollahpour-Alitappeh M, Ehsani P. Biomedical applications of yeast- a patent view, part one: yeasts as workhorses for the production of therapeutics and vaccines. Expert Opin Ther Pat. 2017;27: 929–951. 10.1080/13543776.2017.1339789 28608761

[63] Haim L, Zipor G, Aronov S, Gerst JE. A genomic integration method to visualize localization of endogenous mRNAs in living yeast. Nat Methods. 2007;4: 409–412. 10.1038/nmeth1040 17417645

[64] Storici F, Resnick MA. The Delitto Perfetto Approach to In Vivo Site-Directed Mutagenesis and Chromosome Rearrangements with Synthetic Oligonucleotides in Yeast. Methods Enzymol. 2006;409: 329–345. 10.1016/S0076-6879(05)09019-1 16793410

[65] Ye J, Coulouris G, Zaretskaya I, Cutcutache I, Rozen S, Madden TL. Primer-BLAST: A tool to design target-specific primers for polymerase chain reaction. BMC Bioinformatics. 2012;13: 134 10.1186/1471-2105-13-134 22708584 PMC3412702

[66] Ram AFJ, Klis FM. Identification of fungal cell wall mutants using susceptibility assays based on Calcofluor white and Congo red. Nat Protoc. 2006;1: 2253–2256. 10.1038/nprot.2006.397 17406464

[67] Zhang T, Lei J, Yang H, Xu K, Wang R, Zhang Z. An improved method for whole protein extraction from yeast Saccharomyces cerevisiae. Yeast. John Wiley & Sons, Ltd; 2011;28: 795–798. 10.1002/yea.1905 21972073

[68] Goldstein A, Lampen JO. Beta-D-fructofuranoside fructohydrolase from yeast. Methods Enzymol. 1975;42:504–511. 237205 10.1016/0076-6879(75)42159-0

[69] Novick P, Schekman R. Secretion and cell-surface growth are blocked in a temperature-sensitive mutant of Saccharomyces cerevisiae. Proc Natl Acad Sci U S A; 1979;76: 1858–62. 10.1073/pnas.76.4.1858 377286 PMC383491

[70] Troy AA H, Harkness T. A Simplified Method for Measuring Secreted Invertase Activity in Saccharomyces cerevisiae [Internet]. Biochemistry & Pharmacology: Open Access. 2014 10.4172/2167-0501.1000151

[71] Mueller F, et al FISH-quant: automatic counting of transcripts in 3D FISH images. Nat Methods. 2013;10(4):277–278 10.1038/nmeth.2406 23538861

[72] Sonnhammer EL, von Heijne G, Krogh A. A hidden Markov model for predicting transmembrane helices in protein sequences. Proc Int Conf Intell Syst Mol Biol. 1998;6: 175–182. doi:9783223 9783223

[73] Bailey TL, Boden M, Buske FA, Frith M, Grant CE, Clementi L, et al MEME SUITE: tools for motif discovery and searching. Nucleic Acids Res. 2009;37: W202–W208. 10.1093/nar/gkp335 19458158 PMC2703892

