# Supplementary material for: A secretion-enhancing cis regulatory targeting element (SECReTE) involved in mRNA localization and protein synthesis
Source: PLoS Genet. 2019 Jul 1;15(7):e1008248. doi: 10.1371/journal.pgen.1008248 (PMC6625729; doi:10.1371/journal.pgen.1008248)
Supplement: S1 Table — (DOCX) [file pgen.1008248.s001.docx]

**Table S1. Yeast strains used in this study**

| **Name** | **Genotype** | **Source** |
| --- | --- | --- |
| WT BY4741 | *MAT*a *his3Δ1 leu2Δ0 met15Δ0 ura3Δ0* | Euroscarf |
| *suc2Δ* | *MAT*a *his3Δ1 leu2Δ0 met15Δ0 ura3Δ0 suc2Δ::natMX* | This study |
| *SUC2(-)SECReTE* | *MAT*a *his3Δ1 leu2Δ0 met15Δ0 ura3Δ0 SUC2::SUC2(-)SECReTE* | This study |
| *SUC2(+)SECReTE* | *MAT*a *his3Δ1 leu2Δ0 met15Δ0 ura3Δ0 SUC2::SUC2(+)SECReTE* | This study |
| *hsp150Δ* | *MAT*a *his3Δ1 leu2Δ0 met15Δ0 ura3Δ0 hsp150Δ::natMX* | This study |
| *hsp150(-)SECReTE* | *MAT*a *his3Δ1 leu2Δ0 met15Δ0 ura3Δ0 HSP150::HSP150(-)SECReTE* | This study |
| *hsp150(+)SECReTE* | *MAT*a *his3Δ1 leu2Δ0 met15Δ0 ura3Δ0 HSP150::HSP150(+)SECReTE* | This study |
| *ccw12Δ* | *MAT*a *his3Δ1 leu2Δ0 met15Δ0 ura3Δ0 ccw12Δ::natMX* | This study |
| *CCW12(-)SECReTE* | *MAT*a *his3Δ1 leu2Δ0 met15Δ0 ura3Δ0 CCW12::CCW12(-)SECReTE* | This study |
| *she2Δ* | *MAT*a *his3Δ1 leu2Δ0 met15Δ0 ura3Δ0 she2Δ::natMX* | This study |
| *she2Δ HSP150(+)SECReTE* | *MAT*a *his3Δ1 leu2Δ0 met15Δ0 ura3Δ0 she2Δ::natMX HSP150::HSP150(+)SECReTE* | This study |
| *puf2Δ* | *MAT*a *his3Δ1 leu2Δ0 met15Δ0 ura3Δ0 puf2Δ::natMX* | This study |
| *puf2Δ HSP150(+)SECReTE* | *MAT*a *his3Δ1 leu2Δ0 met15Δ0 ura3Δ0 puf2Δ::natMX HSP150::HSP150(+)SECReTE* | This study |
| *puf1Δ* | *MAT*a *his3Δ1 leu2Δ0 met15Δ0 ura3Δ0 puf1Δ::natMX* | This study |
| *puf1Δ HSP150(+)SECReTE* | *MAT*a *his3Δ1 leu2Δ0 met15Δ0 ura3Δ0 puf1Δ::natMX HSP150::HSP150(+)SECReTE* | This study |
| *whi3Δ* | *MAT*a *his3Δ1 leu2Δ0 met15Δ0 ura3Δ0 whi3Δ::natMX* | This study |
| *whi3Δ HSP150(+)SECReTE* | *MAT*a *his3Δ1 leu2Δ0 met15Δ0 ura3Δ0 whi3Δ::natMX HSP150::HSP150(+)SECReTE* | This study |
| *khd1Δ* | *MAT*a *his3Δ1 leu2Δ0 met15Δ0 ura3Δ0 khd1Δ::natMX* | This study |
| *khd1Δ HSP150(+)SECReTE* | *MAT*a *his3Δ1 leu2Δ0 met15Δ0 ura3Δ0 khd1Δ::natMX HSP150::HSP150(+)SECReTE* | This study |
