# Supplementary material for: A secretion-enhancing cis regulatory targeting element (SECReTE) involved in mRNA localization and protein synthesis
Source: PLoS Genet. 2019 Jul 1;15(7):e1008248. doi: 10.1371/journal.pgen.1008248 (PMC6625729; doi:10.1371/journal.pgen.1008248)
Supplement: S2 Table — (DOCX) [file pgen.1008248.s002.docx]

**Table S2. Plasmids used in this study**

| **Name** | **Copy number** | **Selection marker** | **Origin** |
| --- | --- | --- | --- |
| pRS416-SEC63-GFP | 2µ | *URA3* | J. Gerst |
| pYES2-SSGAS1-GFP-GAS1^3'UTR^(+)SECReTE | 2µ | *LEU2* | This study |
| pYES2-SSGAS1-GFP | 2µ | *LEU2* | This study |
| pYES2-GFP | 2µ | *LEU2* | Ast *et al*., 2013 |
| pYES2-SSKAR-GFP | 2µ | *LEU2* | Ast *et al.,* 2013 |
| pYES2-SSGAS1-LacZ | 2µ | *LEU2* | This study |
