# Supplementary material for: A secretion-enhancing cis regulatory targeting element (SECReTE) involved in mRNA localization and protein synthesis
Source: PLoS Genet. 2019 Jul 1;15(7):e1008248. doi: 10.1371/journal.pgen.1008248 (PMC6625729; doi:10.1371/journal.pgen.1008248)
Supplement: S5 Table — (DOCX) [file pgen.1008248.s005.docx]

**Table S5. SECReTE score alteration in *SUC2, HSP150*, and *CCW12***

|  |  | **SECReTE score and codon position** | | |  |  |
| --- | --- | --- | --- | --- | --- | --- |
| **Name** | **Length** | ***YNN*** | ***NYN*** | ***NNY*** | **Free energy** | **CAI** |
| *SUC2* | 1599 | 0 | 0 | 1 | -414.8 | 0.79 |
| *SUC2*(+)SECReTE | 1599 | 0 | 0 | 6 | -405.7 | 0.8 |
| *SUC2*(-)SECReTE | 1599 | 0 | 0 | 0 | -408.7 | 0.72 |
| *HSP150* | 1242 | 0 | 4 | 2 | -312.7 | 0.83 |
| *HSP150*(+)SECReTE | 1242 | 0 | 4 | 4 | -297 | 0.84 |
| *HSP150*(-)SECReTE | 1242 | 0 | 4 | 0 | -358.5 | 0.8 |
| *CCW12* | 402 | 0 | 3 | 3 | -85.3 | 0.83 |
| *CCW12*(-)SECReTE | 402 | 0 | 3 | 1 | -106.9 | 0.8 |
