# Supplementary figures and images for: A secretion-enhancing cis regulatory targeting element (SECReTE) involved in mRNA localization and protein synthesis
Source: PLoS Genet. 2019 Jul 1;15(7):e1008248. doi: 10.1371/journal.pgen.1008248 (PMC6625729; doi:10.1371/journal.pgen.1008248)

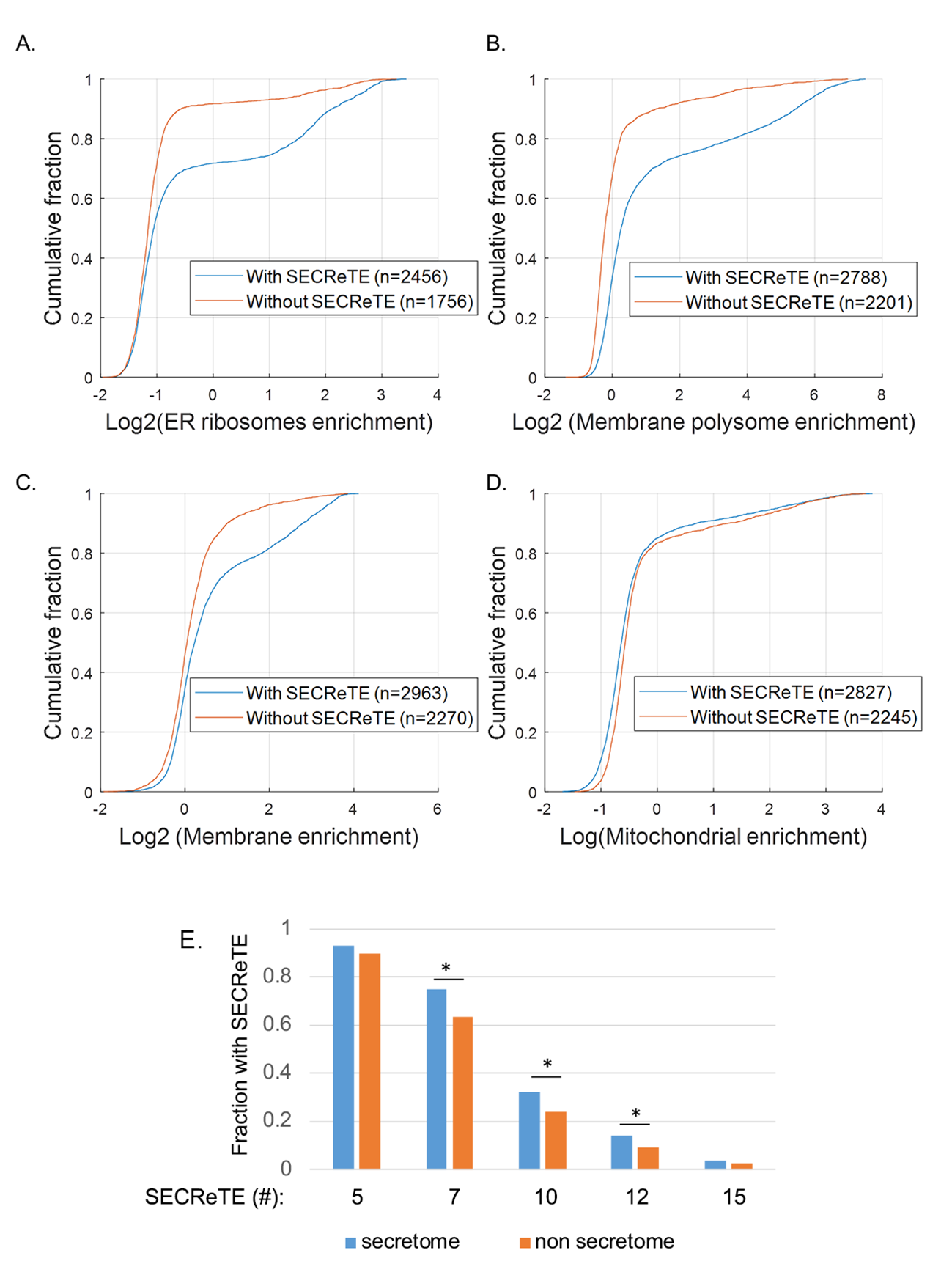

Supplement: S1 Fig — Cumulative distribution of mRNA enrichment was plotted separately for SECReTE-containing transcripts and transcripts lacking SECReTE. (A) Transcripts containing SECReTE are more abundant on ER-bound ribosomes. A plot of the enrichment data obtained from proximity-specific ribosome profiling with BirA-Ubc6 (2min; cycloheximide–CHX) [22]. (B) Transcripts with SECReTE are more abundant on membranal polysomes. A plot of the enrichment data obtained from the ribosome profiling of polysomes extracted from the membrane fraction of yeast cells [23]. (C) Transcripts with SECReTE are more abundant in the membrane fraction. A plot of the enrichment data obtained from RNA-seq analysis of the membrane fraction of yeast cells [23]. (D) Transcripts with SECReTE are not abundant on mitochondrial ribosomes. A plot of the enrichment data obtained from proximity-specific ribosome profiling with BirA-Om45 (2min; CHX) [22]. (E) Removal of the TMD regions does not alter the enrichment of SECReTE motifs in mRNAs coding for TMD-containing secretome proteins than for non-secretome TMD-containing proteins. SECReTE presence, according to the indicated threshold, was counted in mRNAs coding for TMD-encoding secretome (blue) and non-secreted (orange) proteins in which the TMD regions were first removed. Bars represent the fraction of SECReTE positive transcripts at the indicated threshold. SECReTE abundance remains significantly higher in the TMD-encoding secretome mRNAs. *p < 0.008 (secretome vs. non-secretome; SECReTE7); <0.015 (SECReTE10); <0.05 (SECReTE12), chi-square. (TIF) [file pgen.1008248.s006.tif]

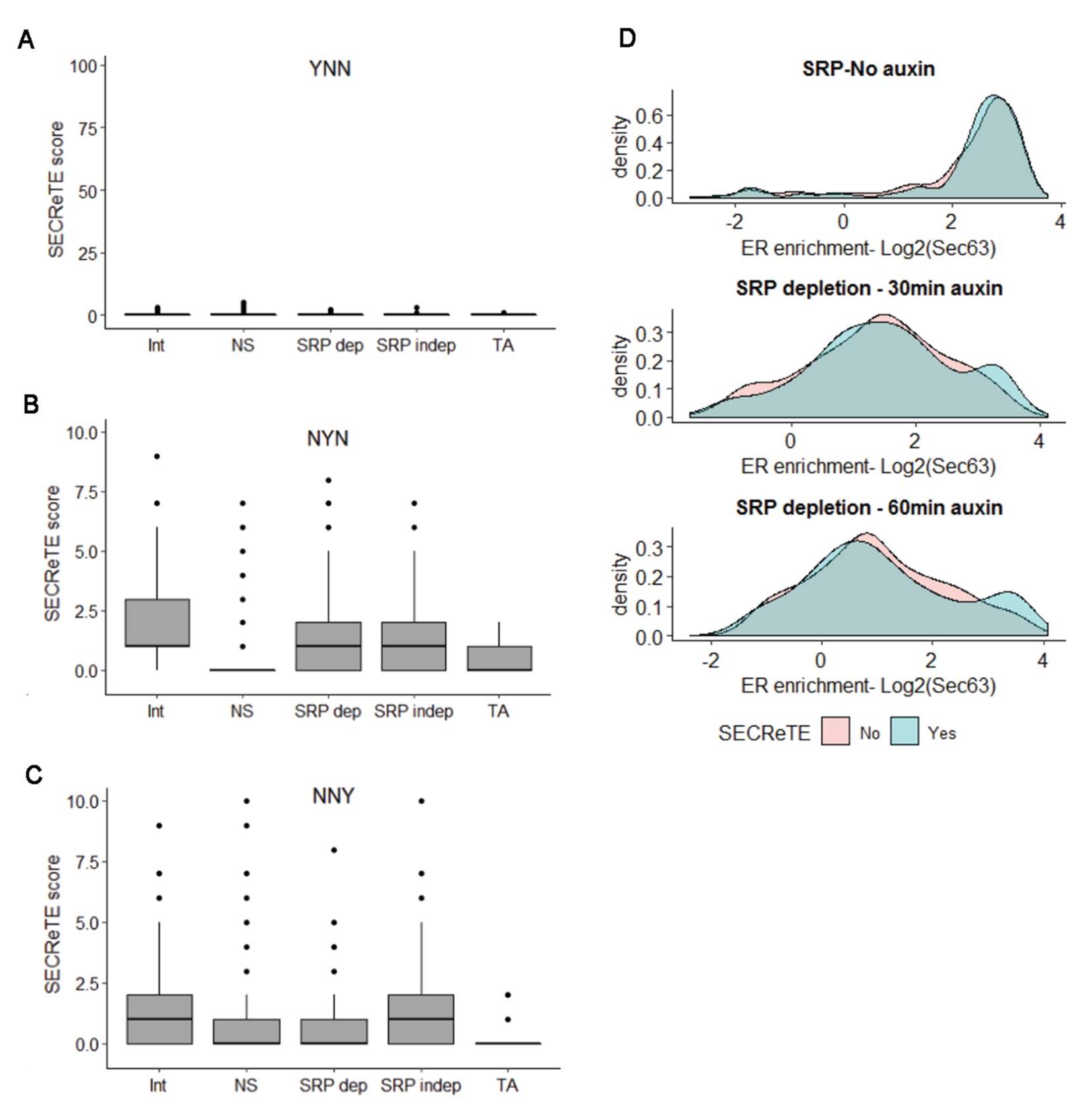

Supplement: S2 Fig — (A-C) SECReTE10 distribution in groups of genes separated according to their SRP-dependence predictions. Genes were separated according to prediction of their SRP-dependence [19] in the dataset of Costa et al [24]. Box-plots represent the distribution of SECReTE10 in each of the coding positions in the different groups: Int—internal TMD; NS–non-secretome; SRP dep–SRP-dependent; SRP indep–SRP-independent; TA—tail- anchored. (A) SECReTE10 distribution at the first codon position. As was shown, SECReTE10 is barely present in the first codon position of all genes. (B) SECReTE10 distribution at the second codon position. Secretome groups are enriched with SECReTE10 at the second codon position. Among secretome genes those with an internal TMD (Int) are the most enriched, while tail-anchored genes are the least enriched. (C) SECReTE10 distribution at the third codon position. SECReTE10 at the third position is more enriched in SRP-independent genes (SRP indep) than in SRP-dependent genes (SRP dep). Genes with an internal TMD (Int) are also enriched with SECReTE10 at the third position of the codon. (D) SECReTE is enriched in mRNAs that are ER-bound after SRP-depletion. Secretome transcripts were separated according to SECReTE10 presence or absence at the third position of the codon upon auxin-induced SRP depletion. SECReTE10-containing genes = light blue; SECReTE10-lacking genes = pink. Density plots were obtained from data of a time course of SRP depletion using proximity-specific ribosome profiling with Sec63-BirA in the presence of CHX. (E-F) mRNAs encoding cell wall proteins remain ER-bound after SRP-depletion. Cell component ontology analysis revealed that transcripts remaining on the ER after 60min of SRP depletion [log2(Sec63-BirA)>2] are highly enriched with cell wall proteins (enrichment is 2.8-fold higher than mSMPs (E) and 14.3-fold higher than all yeast genes (F). (TIF) [file pgen.1008248.s007.tif]

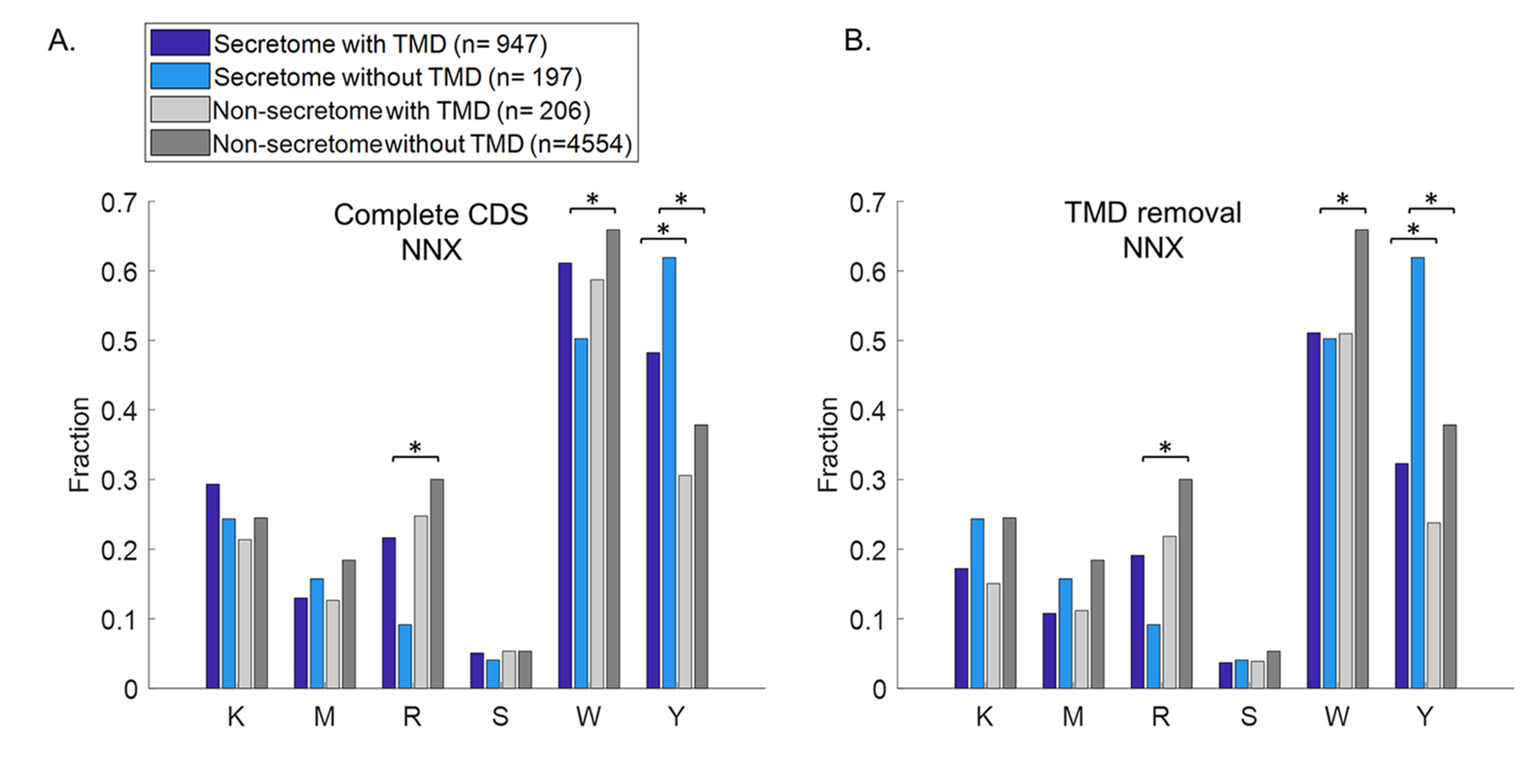

Supplement: S3 Fig — Computational analysis of ≥10 repetitive NNX motifs in the coding region (CDS) of secretome and non-secretome transcripts, either with (A) or without (B) the transmembrane domains (TMD), respectively, is shown. X = K (T/G), M (C/A), R (A/G), S (G/C), or W (A/T)]. NNR and NNW motifs are significantly more abundant in non-secretome genes without transmembrane domains (p = 1.3e-9 and 1.8e-5, chi-square after false discovery rate correction, respectively). (TIF) [file pgen.1008248.s008.tif]

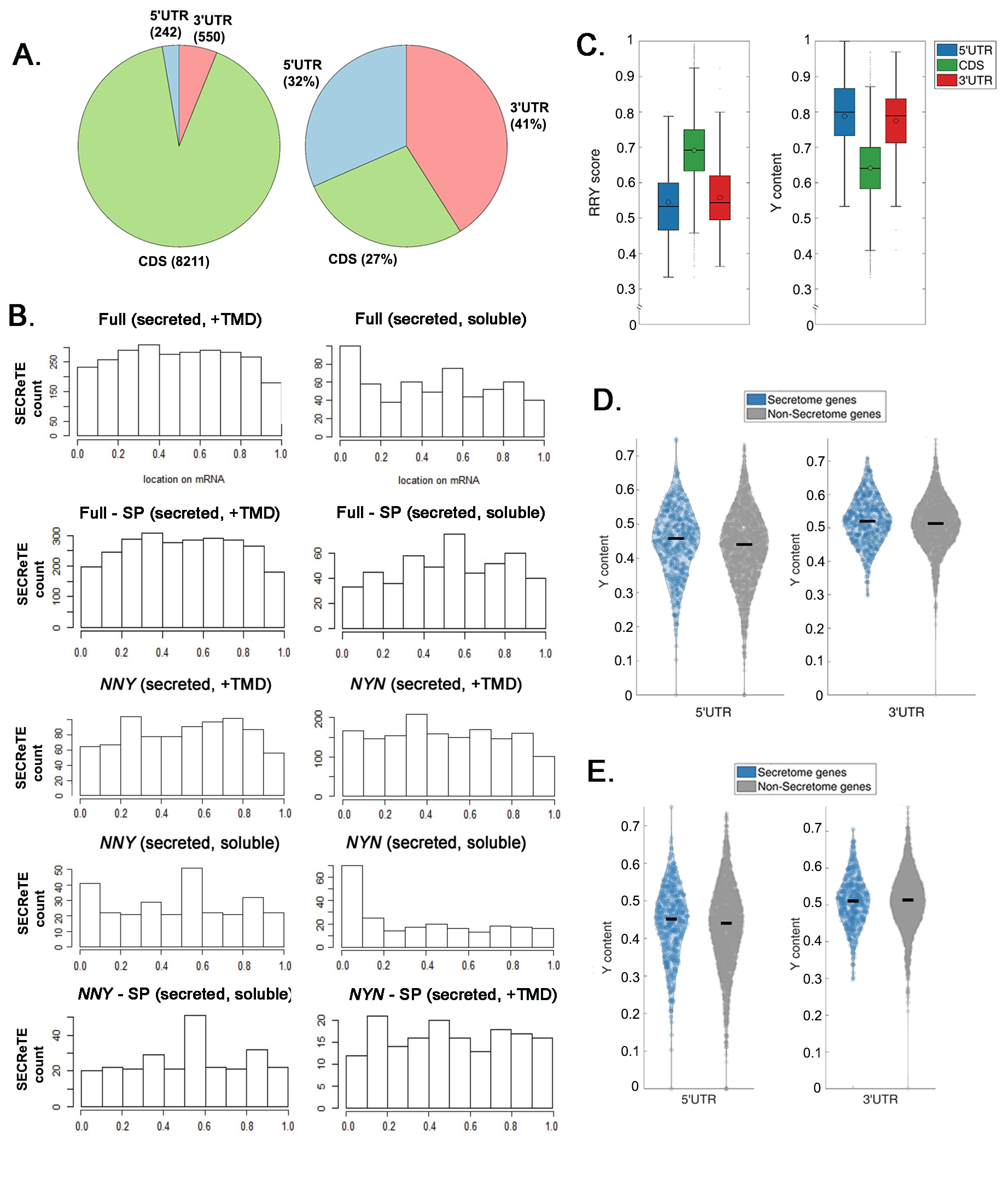

Supplement: S4 Fig — SECReTE distribution and content (A) SECReTE is evenly distributed over the coding and UTR regions in yeast. SECReTE distribution was plotted over the different gene regions (e.g. CDS, 5’UTR, and 3’UTR) of the yeast (left). Most SECReTE motifs (i.e. ≥10 NNY) are found in the CDS (left). However, normalization of SECReTE abundance over the mean region length shows that SECReTE is more or less evenly distributed between regions. (B) SECReTE is uniformly distributed in genes expressing TMD-containing secretome proteins, but is somewhat more prevalent at the 5’ end of genes encoding soluble secretome proteins. SECReTE motif (i.e. ≥10) distribution along the length of genes encoding secretome proteins was scored for both soluble and TMD-containing proteins before and after removal of the signal sequence-encoding region (SSCR:—SP). Count = number of genes with motif in delimited region. Location = location of motif along normalized gene length. Top four graphs illustrate SECReTE distribution in the full length genes including the UTR regions (Full). Bottom six graphs show the SECReTE count scored according to frame (i.e. NNY and NYN) in the coding regions, either before or after removal of the SSCR (- SP). The results show that SECReTE is uniformly distributed in TMD-containing proteins, but that the SSCR can contribute the motif in a subset of ~60 genes encoding soluble secreted proteins. (C) SECReTE motifs in the CDS follow an RRY pattern, while UTR motifs are pyrimidine-rich. The RRY score (see Methods) of SECReTE (≥10 NNY) residing in the CDS and UTRs of yeast genes were determined. RRY-based SECReTE motifs are significantly higher than those residing in the UTR (left) (unpaired t-test, p value <10−25). The pyrimidine content of SECReTE in the gene regions was also scored (right) and the UTR-based motifs show a higher Y content (unpaired t-test,p-value < 10−25). (D) UTRs of secretome-encoding genes are enriched with pyrimidine compared to non-secretome-encoding genes. [file pgen.1008248.s009.tif]

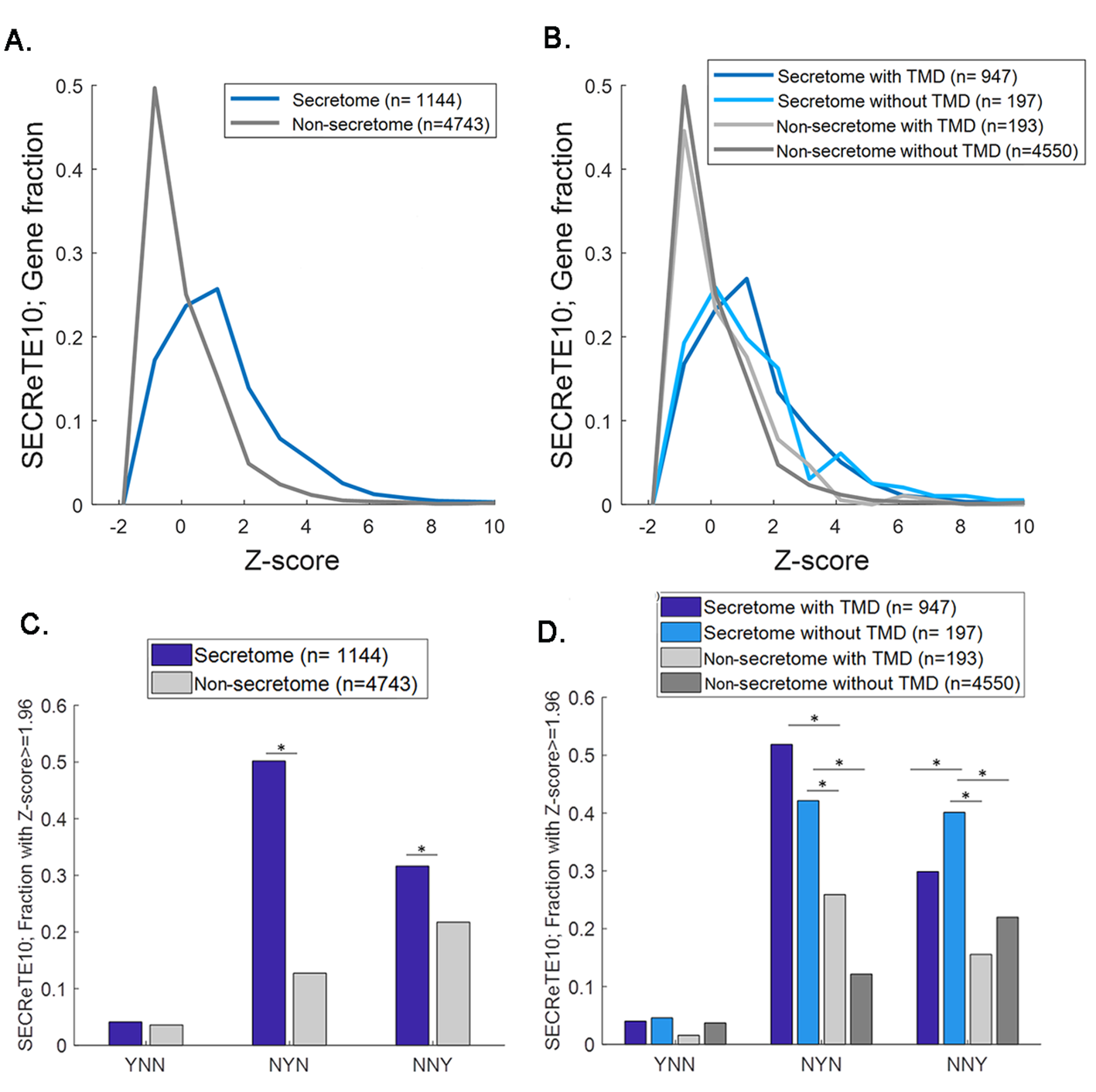

Supplement: S5 Fig — Permutation analysis was conducted to evaluate the dependency of SECReTE on codon usage. To do that, codon composition was kept and sequences were randomly reshuffled 1000 times. The Z-score was calculated for each gene to assess the probability of the SECReTE10 to appear randomly (for Z-score calculation, see Materials and Methods). The higher the Z-score the less likely it is for SECReTE to appear randomly. (A) SECReTE enrichment in secretome-encoding mRNAs is independent of codon usage. Distribution plots of Z-scores show higher values for mRNAs encoding secretome proteins than for non-secretome proteins. (B) SECReTE enrichment in mRNAs encoding both soluble and membranal secretome transcripts is independent of codon usage. Distribution plots of Z-scores show higher values for mRNAs encoding secretome proteins (mSMPs; either with or without a TMD) than for non-secretome proteins (i.e. with or without a TMD). (C) SECReTE enrichment in the second and third position of the codon is independent of codon usage. The fraction of significant Z-scores (i.e. ≥1.96) is larger for mRNAs encoding secretome proteins than for non-secretome proteins. (D) SECReTE enrichment in the second and third position of the codon is independent of both codon usage and TMD presence. The fraction of significant Z-scores (i.e. ≥1.96) is larger for mRNAs encoding secretome proteins than for non-secretome proteins, either with or without a TMD. (TIF) [file pgen.1008248.s010.tif]

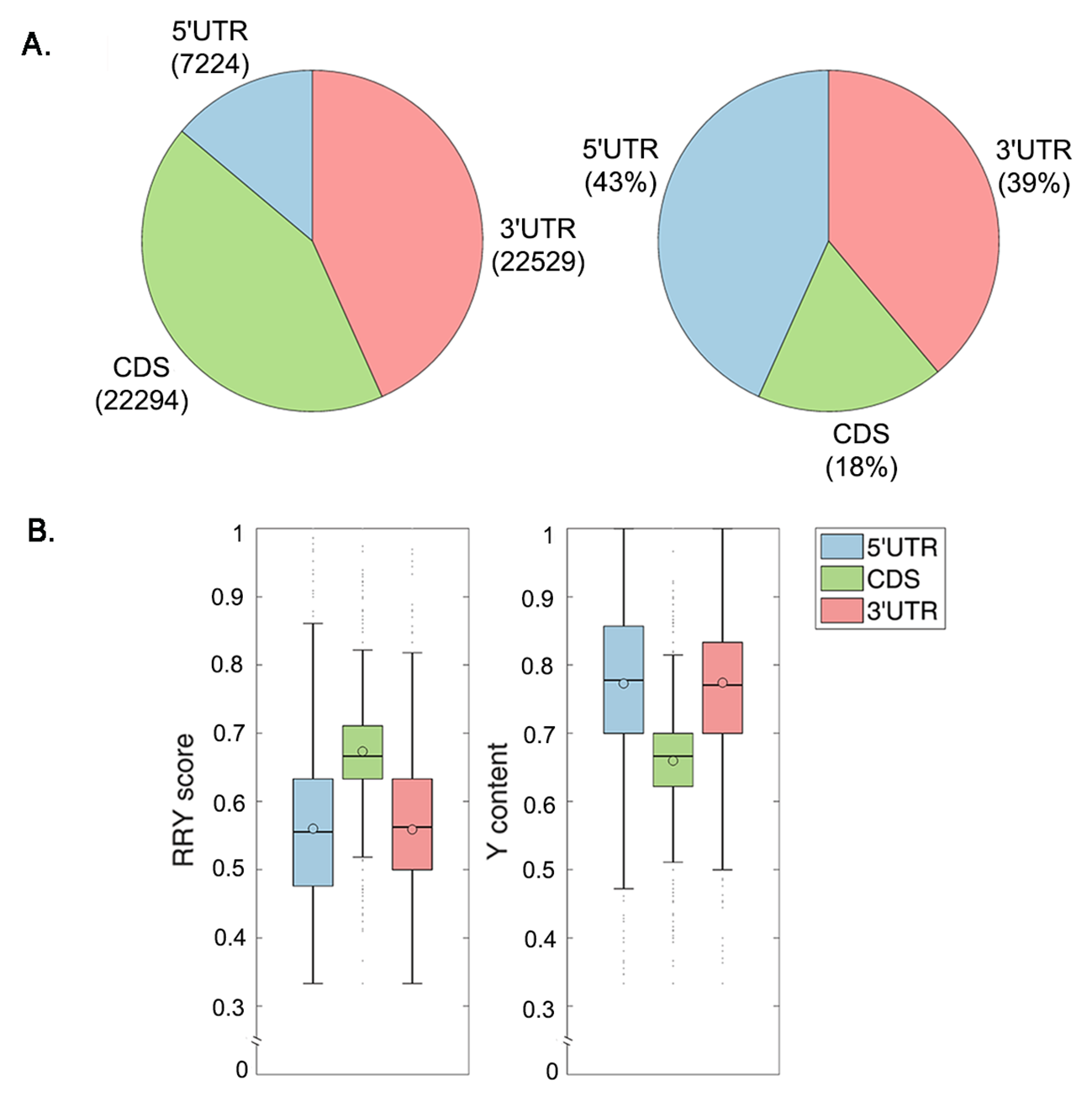

Supplement: S6 Fig — A) Human SECReTE motifs are mainly UTR-localized. Computational analysis of SECReTE (≥10 NNY) distribution over the different gene regions [5’UTR, 3’UTR, and coding region (CDS)] of humans, either without (left) or with (right) normalization for region length (based upon mean length of the region) is shown. (B) Human SECReTE motifs in the CDS follow the RRY pattern, while the UTRs are pyrimidine-rich. Computational analysis of RRY (≥10 RRY) enrichment (left) or pyrimidine content (right) for the different gene regions is shown. RRY repeat scoring (see Methods) of SECReTE motifs residing in the CDS is significantly higher than that of UTR-based SECReTE motifs (left; unpaired t-test, p value < 10−100). Yet, UTR-based motifs have a significantly higher Y-content (right; unpaired t-test, p value < 10−100). (TIF) [file pgen.1008248.s011.tif]

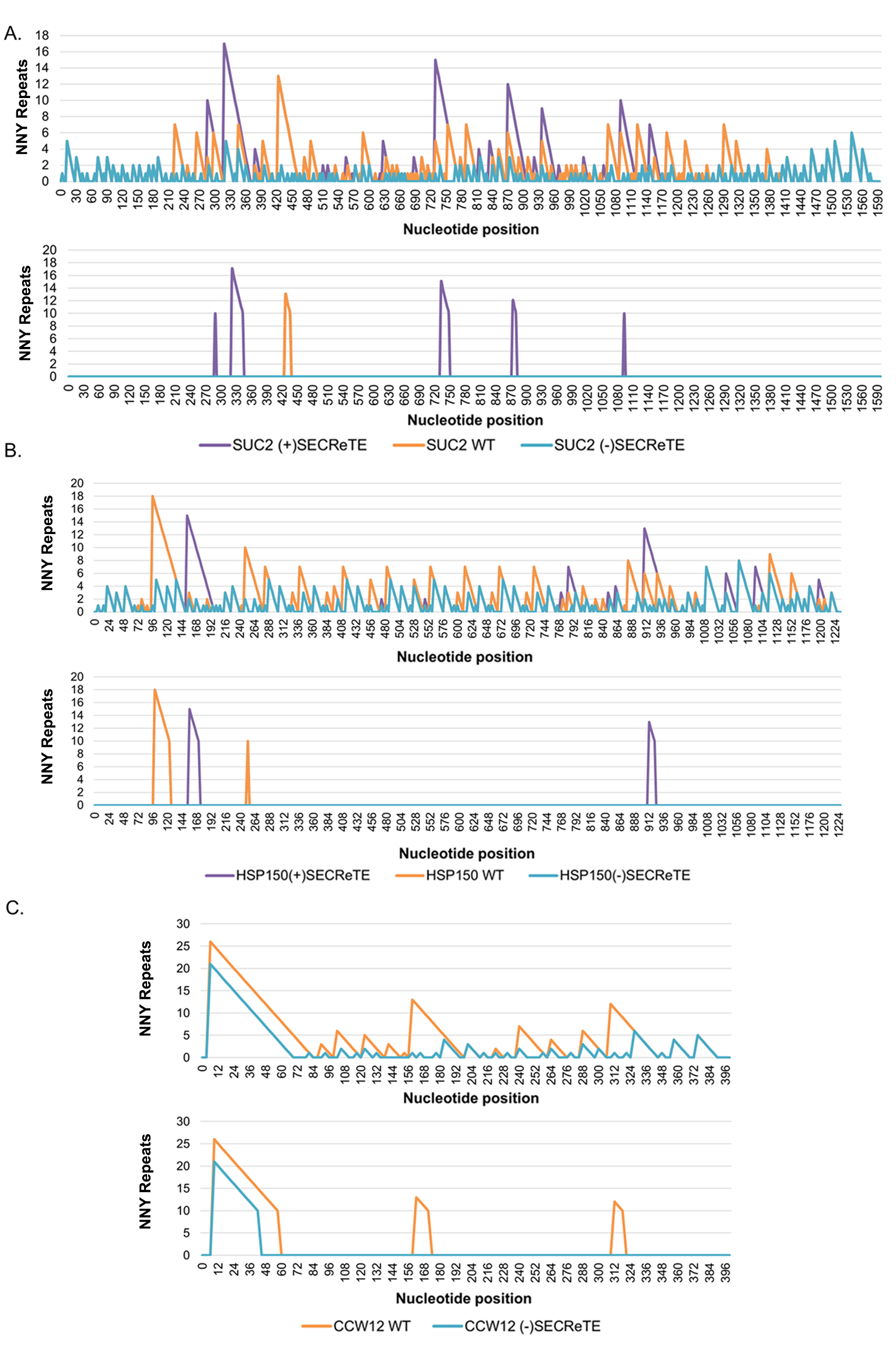

Supplement: S7 Fig — Graphs compare the number of triplet NNY repeats found along the length of the coding region of each gene either with (lower schematics) or without using a threshold of 10 consecutive NNY repeats (upper schematics) in the native and mutant SECReTE genes. (A) SUC2. (B) HSP150. (C) CCW12. (TIF) [file pgen.1008248.s012.tif]

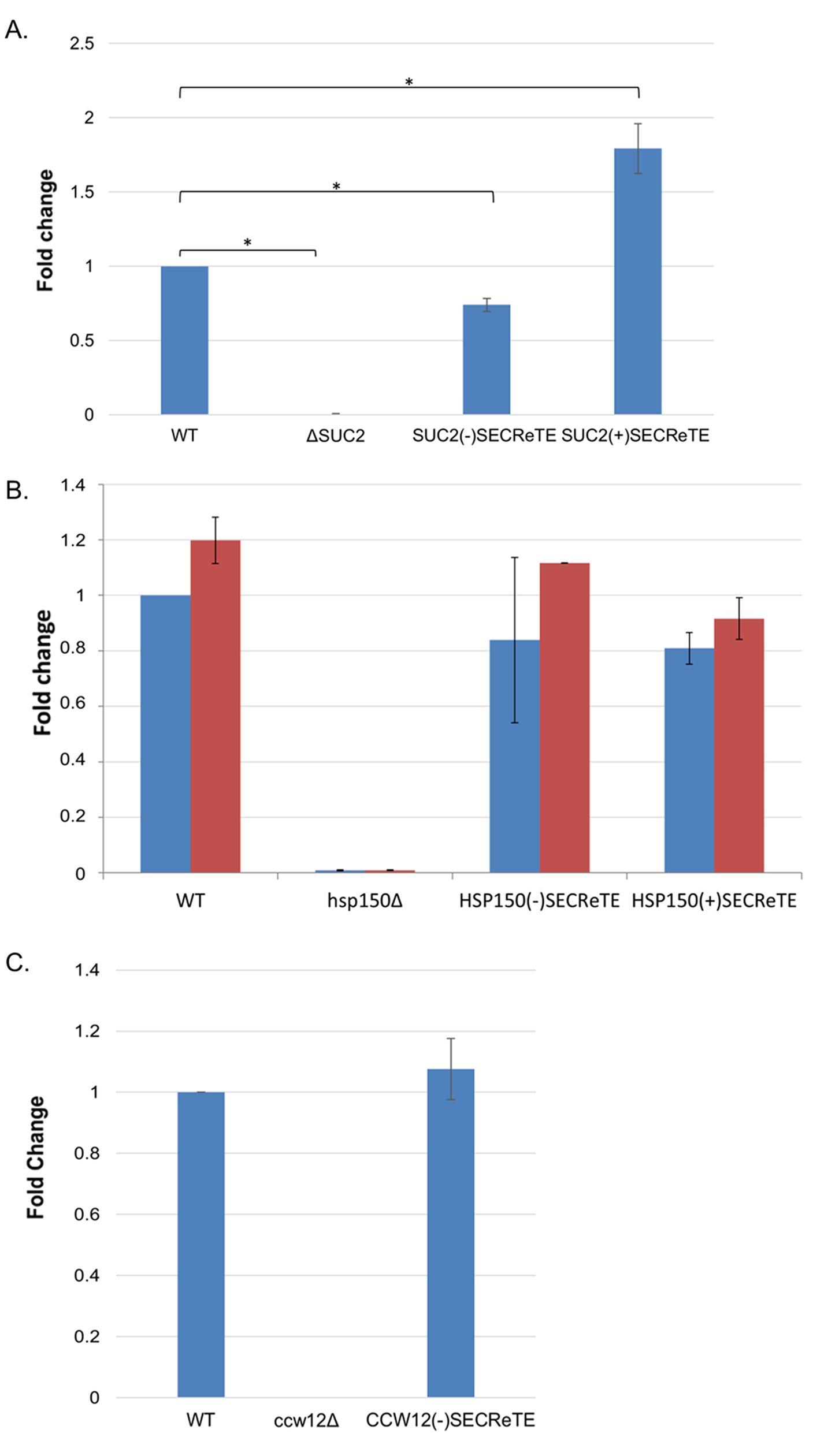

Supplement: S8 Fig — mRNA levels of native or mutant SUC2, CCW12, and HSP150 in the indicated strains were quantified by qRT-PCR. Fold-change was calculated relative to WT levels. (A) SUC2 mRNA levels are altered by SECReTE mutation. Cells were grown to mid-log phase on SC medium containing 2% glucose at 30°C prior to shifting cells to low glucose medium for 1.5hrs. After harvesting and RNA extraction, primers used for amplifying the long transcript of SUC2, which encodes the secreted protein. Primers for actin were used for normalization. SUC2(-)SECReTE cells exhibited lower SUC2 mRNA levels than WT, while SUC2(+)SECReTE cells yielded higher levels. Error bars represent the standard deviation of three biological repeats. (B) HSP150 mRNA levels are not altered by SECReTE mutation. Yeast strains were grown to mid-log phase at either 26°C (red) or 37°C (blue) on YPD medium prior to harvesting and RNA extraction. UBC6 was used for normalization. HSP150 mRNA levels were not significantly changed as a result of SECReTE alterations. (C) CCW12 mRNA levels are not altered by SECReTE mutation. Cells were grown to mid-log phase onYPD medium at 30°C prior to harvesting and RNA extraction. Primers used for amplifying UBC6 were used for normalization. CCW12 mRNA levels were not significantly changed as a result of SECReTE alterations. (TIF) [file pgen.1008248.s013.tif]

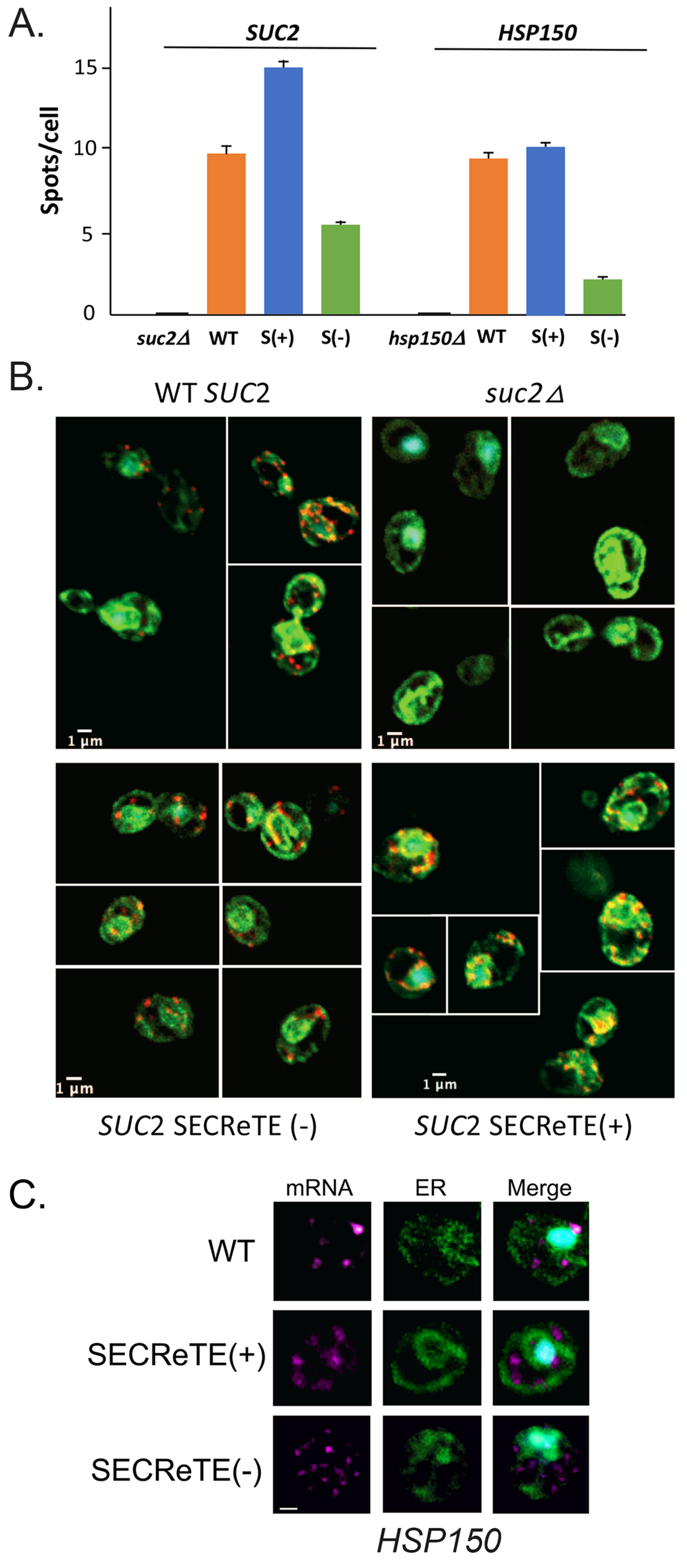

Supplement: S9 Fig — (A) mRNA granule scores per cell measured using FISH-quant. The number of mRNA granules (spots) detected by smFISH using Stellaris probes for SUC2 and HSP150 was scored for WT, (+)SECReTE (S+) and (-)SECReTE (S-) cells using FISH-quant (https://bitbucket.org/muellerflorian/fish_quant). The numbers correspond to the average number of mRNAs detected per cell ±SEM for the data shown in Fig 6A and 6B, and S9B and S9C Fig. (B) Visualization of endogenously expressed SUC2(+)SECReTE and SUC2(-)SECReTE mRNAs using smFISH. Yeast endogenously expressing WT SUC2, SUC2(+)SECReTE, or SUC2(-)SECReTE and Sec63-GFP from a plasmid were grown to mid-log phase on SC medium containing 2% glucose prior to shifting cells to low glucose-containing medium (0.05% glucose) to induce SUC2 expression. Cells were processed for smFISH labeling using non-overlapping, TAMRA-labeled, FISH probes complementary to SUC2, prior to labeling with DAPI, as shown in Fig 6A. Additional representative images are shown in this figure. Line = 1μm. (C) Visualization of endogenously expressed HSP150(+)SECReTE and HSP150(-)SECReTE mRNAs using smFISH. Yeast endogenously expressing WT HSP150, HSP150(+)SECReTE, or HSP150(-)SECReTE and Sec63-GFP from a plasmid were grown to mid-log phase on SC medium prior to fixation. Cells were processed for smFISH labeling using non-overlapping, TAMRA-labeled, FISH probes complementary to HSP150, and stained with DAPI (shown in merge). mRNA granule scoring was performed using the FISH-quant algorithm and co-localization of the granules to the ER (both cER and nER) is shown in Fig 6B. (TIF) [file pgen.1008248.s014.tif]

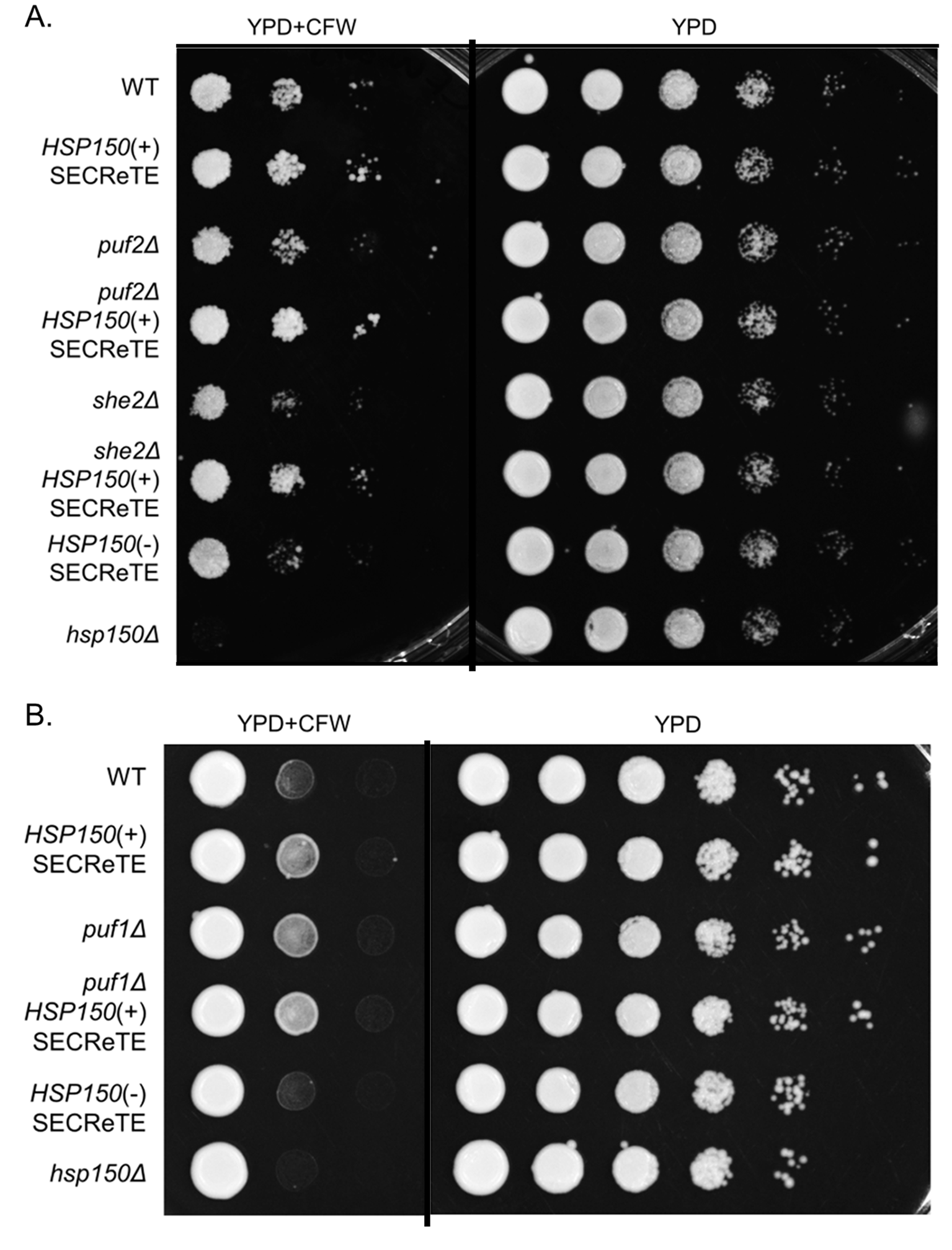

Supplement: S10 Fig — WT cells and either WT or HSP150(+)SECReTE cells deleted for genes encoding the indicated RBPs [e.g. Puf2, She2 (A) and Puf1(B)] were grown to mid-log phase on YPD at 30°C, prior to serial dilution and plating onto either solid YPD medium or YPD containing CFW. Yeast were grown 2 days prior to photo-documentation. (TIF) [file pgen.1008248.s015.tif]
